# Advances in Somatostatin Receptor-Targeting Peptide Receptor Radionuclide Therapy for Neuroendocrine Tumors: Biological Rationale, Combination Strategies, and Clinical Perspectives

**DOI:** 10.3390/ijms27198709

**Published:** 2026-09-29

**Authors:** Hana Bakr, Amin Al-Awar, Johan K. E. Spetz, Eva B. Forssell-Aronsson

**Affiliations:** 1 Department of Medical Radiation Sciences, Institute of Clinical Sciences, Sahlgrenska Academy, University of Gothenburg, SE-413 45 Gothenburg, Sweden; hana.bakr@gu.se (H.B.); amin.al-awar@bcm.edu (A.A.-A.); johan.spetz@gu.se (J.K.E.S.); 2 Sahlgrenska Center for Cancer Research, Sahlgrenska Academy, University of Gothenburg, SE-405 30 Gothenburg, Sweden; 3 Department of Medical Physics and Biomedical Engineering, Sahlgrenska University Hospital, Region Västra Götaland, SE-413 45 Gothenburg, Sweden

**Keywords:** lutetium-177, radiosensitization, somatostatin receptor upregulation, lutathera, radiopharmaceuticals, personalized dosimetry, theranostics, DNA damage repair, treatment optimization, targeted radionuclide therapy

## Abstract

Gastroenteropancreatic (GEP) neuroendocrine tumors (NETs) are the most common subtype of NETs, and their incidence continues to increase worldwide. Many NETs express somatostatin receptors (SSTRs), enabling molecular imaging and treatment with radiolabeled somatostatin analogs (SSTAs). [^177^Lu]Lu-DOTATATE is an established treatment for advanced SSTR-positive NETs, but complete and durable responses remain uncommon. This review summarizes current clinical experience with [^177^Lu]Lu-DOTATATE and emerging SSTR-targeting radiopharmaceuticals and examines strategies to improve PRRT efficacy. Treatment may be enhanced at several interconnected steps, including increasing receptor availability and tumor targeting, improving radiopharmaceutical uptake and absorbed dose, preventing repair of radiation-induced damage, suppressing cellular stress adaptation and survival, and promoting tumor cell elimination. These approaches include radiation priming and fractionation, somatostatin analog pretreatment, epigenetic modifiers, inhibitors of DNA repair and cellular survival pathways, immunotherapy, and chemotherapy-based combinations. PARP, DNA-PK, and HSP90 inhibitors and chemotherapy-based combinations have shown particularly strong preclinical or clinical evidence, whereas mTOR inhibitors, tyrosine kinase inhibitors, Hedgehog inhibitors, immunotherapy, NAMPT inhibitors, p53 stabilization, and epigenetic modifiers remain under investigation. Ongoing development of novel radiopharmaceuticals, personalized dosimetry, retreatment strategies, and rational combinations is expected to refine PRRT and improve outcomes for patients with NETs.

## 1. Introduction

The incidence of neuroendocrine tumors (NETs) has increased substantially during recent decades and is currently estimated at approximately 6–7 new cases per 100,000 persons annually in North America and Europe [1,2,3]. Surgery remains the primary curative treatment option; however, many patients present with locally advanced or metastatic disease at diagnosis, precluding curative resection [2,4].

NETs are a heterogeneous group of neoplasms that can arise in multiple organs, most commonly in the gastroenteropancreatic (GEP) tract and lungs [3]. They vary considerably with respect to biological behavior, differentiation status, hormone production, and clinical presentation. NETs may be functioning, resulting in hormone-related syndromes and reduced quality of life, or non-functioning, where symptoms are primarily related to tumor burden and metastatic spread [5,6].

After diagnosis, patients with GEP-NETs are usually first treated with surgery when feasible. In patients with advanced or metastatic disease, other locoregional treatments such as radiofrequency ablation and hepatic intra-arterial interventions may be used together with systemic therapies including somatostatin analogs (SSTAs), chemotherapy, targeted therapies such as the mTOR inhibitor everolimus and the tyrosine kinase inhibitor sunitinib, and peptide receptor radionuclide therapy (PRRT) using radiolabeled SSTAs [5,6,7].

SSTRs are key targets for treatment with radiolabeled SSTAs. [^177^Lu]Lu-DOTATATE therapy is a systemic molecular radionuclide treatment that delivers radiation selectively to SSTR-expressing tumor cells. The radionuclide ^177^Lu emits β-particles with a relatively short tissue penetration range, enabling targeted irradiation of tumor tissue, while concomitant γ-emissions allow post-treatment imaging and dosimetric evaluation. These properties make [^177^Lu]Lu-DOTATATE well suited for theranostic applications, combining targeted therapy with molecular imaging and treatment monitoring [8,9,10]. In recent years, [^177^Lu]Lu-DOTATATE has become an established treatment option for patients with advanced metastatic NETs. Clinical studies have demonstrated prolonged progression-free survival (PFS), durable disease control, and favorable long-term outcomes, leading to regulatory approval by both the EMA and FDA in 2018 [11,12,13]. The clinical outcome of [^177^Lu]Lu-DOTATATE therapy has been relatively successful, with prolonged overall survival (OS) and PFS, although complete responses and cures remain uncommon [3,14]. Adverse effects have been observed in a limited number of patients and are primarily related to bone marrow and kidney toxicity [9,15,16].

In parallel with the clinical implementation of [^177^Lu]Lu-DOTATATE, several new SSTR-targeting radiopharmaceuticals have been developed, including alternative agonists, receptor antagonists, albumin-binding radiopharmaceuticals, and alpha-emitting compounds [17,18,19,20,21,22,23,24,25]. Ongoing clinical studies are also evaluating new treatment indications, personalized dosimetry approaches, retreatment strategies, and novel radiopharmaceutical platforms [20,26,27,28,29,30,31,32,33].

**Figure 1:**
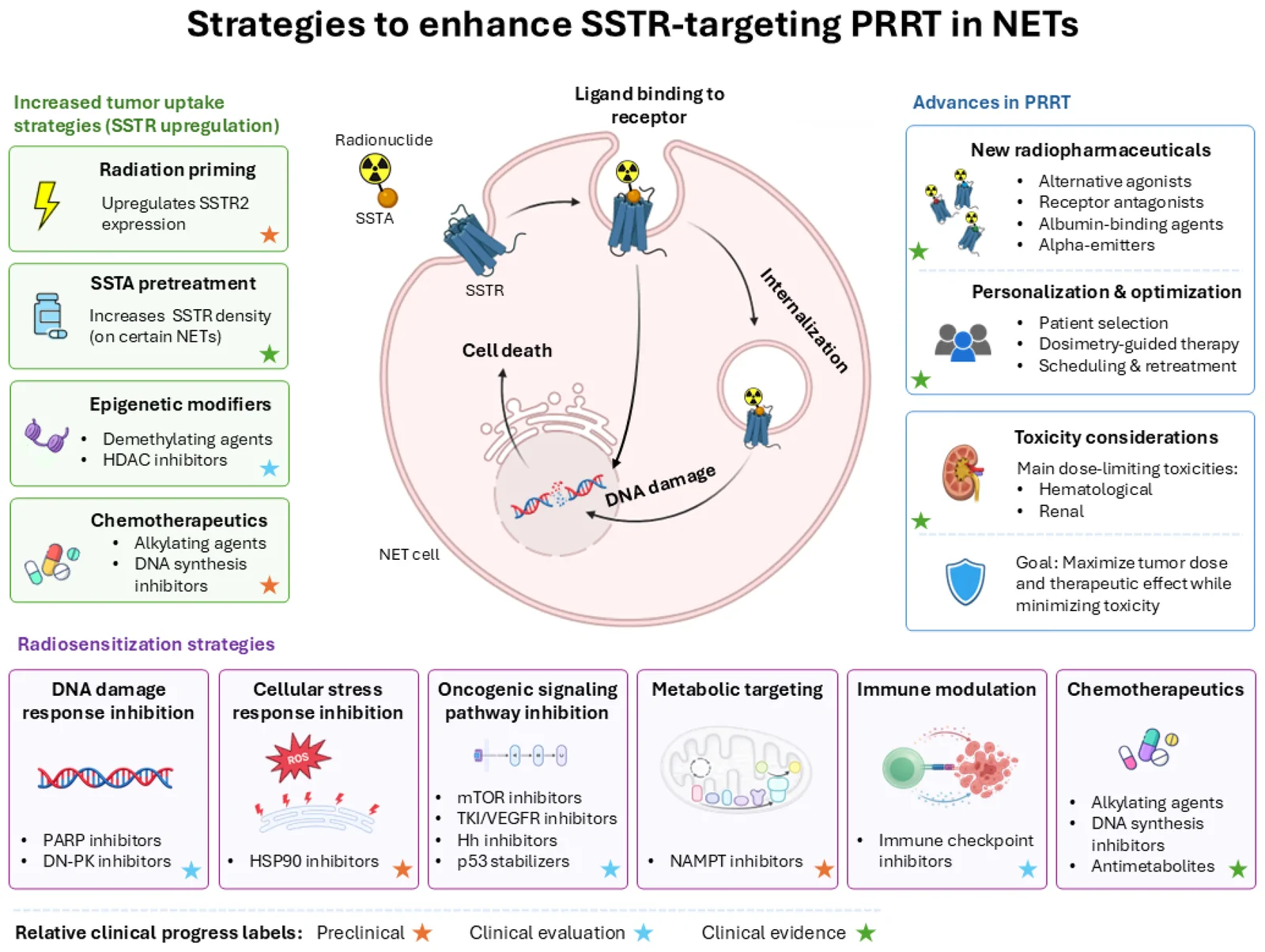
Overview of strategies to enhance SSTR-targeting peptide receptor radionuclide therapy (PRRT) in neuroendocrine tumors. The therapeutic effect of PRRT depends on several interconnected steps, including receptor availability and tumor targeting, radiopharmaceutical uptake and absorbed dose, induction and repair of radiation damage, cellular stress adaptation and survival, and tumor cell elimination. Strategies discussed in this review may act at one or several of these steps. The approximate translational maturity of each strategy is indicated as preclinical, clinical evaluation, or clinical evidence. The figure also highlights emerging developments in PRRT. Together, these approaches aim to improve therapeutic efficacy while maintaining an acceptable safety profile. Image generated using material from BioRender (www.biorender.com).

To further optimize [^177^Lu]Lu-DOTATATE therapy, new strategies to increase the therapeutic effect in tumor tissue while preserving normal tissues need to be developed and evaluated. Broadly, these approaches include strategies that increase tumor uptake of radiolabeled SSTAs and absorbed dose through modulation of SSTR expression, radiopharmaceutical delivery, or treatment scheduling, and strategies that enhance the biological response to radiation [3,7,34]. Several approaches have been investigated, including inhibition of DNA damage repair, cellular stress response and survival pathways, and promotion of tumor cell elimination [3,7]. The principal strategies discussed in this review and their proposed mechanisms for enhancing SSTR-targeting PRRT are summarized in Figure 1.

The aims of this work are to summarize the current clinical and preclinical knowledge regarding PRRT in NETs and to review strategies aimed at improving the efficacy of [^177^Lu]Lu-DOTATATE therapy. We discuss clinical experience with currently available radiopharmaceuticals, ongoing clinical studies, and approaches to increase tumor targeting and absorbed dose through SSTR modulation, radiopharmaceutical delivery, treatment scheduling, and individualized dosimetry. Furthermore, we review preclinical and clinical studies investigating strategies to enhance the biological response to radiation through inhibition of DNA repair, suppression of cellular stress adaptation and survival, and promotion of tumor cell elimination. Finally, we discuss emerging targets and future directions that may contribute to the next generation of PRRT for patients with NETs.

The primary focus of this narrative review is the optimization of treatment of NETs using ^177^Lu-labeled SSTAs. However, when relevant, studies involving other radionuclides, alternative forms of radiation, or other tumors with neuroendocrine properties or SSTR expression are also included to provide biological and translational context. This review was based on literature searches in PubMed and Google Scholar, supplemented by searches of ClinicalTrials.gov and other clinical trial registries, including the EU Clinical Trials Register/EudraCT and the Clinical Trials Registry–India (CTRI). Searches were performed iteratively using combinations and variations in terms related to PRRT and NETs, including *lutetium*, *Lutathera*, *GEP-NET*, and *radiosensitization*, together with terms related to the individual treatment strategies, molecular targets, and radiopharmaceuticals discussed in this review. Additional studies were identified from the reference lists of original research articles and relevant review articles. Preclinical and clinical studies were selected based on their relevance to SSTR-targeted radionuclide therapy and strategies with potential to modify tumor targeting, radiopharmaceutical uptake and absorbed dose, radiation response, or therapeutic efficacy. Given the broad and evolving nature of the field, the literature search was intentionally inclusive and was not designed as a systematic review. Clinical trial registries were additionally searched for ongoing and recently completed studies relevant to SSTR-targeted radionuclide therapy. The literature search was last updated on 12 September 2026.

## 2. Somatostatin and Somatostatin Receptors

Somatostatin (SST) is a peptide hormone that inhibits the secretion of several endocrine and exocrine hormones, including growth hormone (somatotropin). SST exists in two biologically active forms consisting of 14 and 28 amino acids and is widely expressed throughout the neuroendocrine and central nervous systems. In addition to its endocrine functions, SST influences neurotransmission, cellular proliferation, and apoptosis [6,35]. SST exerts its biological effects through somatostatin receptors (SSTRs), which are G protein-coupled receptors expressed on the cell membrane [8]. Five SSTR subtypes (SSTR1-5) have been identified in humans, of which SSTR2 is the most frequently expressed subtype in NETs and the principal target for imaging and therapy with radiolabeled SST analogs [36]. Approximately 80% of NETs express SSTRs at levels suitable for diagnostic imaging and targeted radionuclide therapy [8].

Because endogenous SST has a plasma half-life of only a few minutes, more stable SSTAs have been developed for clinical use. Octreotide (TOC), an octapeptide analog of SST, was approved by the FDA in 1988 for symptom control in patients with functional NETs through suppression of hormone secretion [6]. Other clinically approved SSTAs include lanreotide and pasireotide. Octreotate (TATE) is a closely related octapeptide analog that differs from TOC by a terminal threonine residue, resulting in higher affinity for SSTR2 [37]. Both TOC and TATE derivatives have been extensively used for molecular imaging and peptide receptor radionuclide therapy, although TATE-based compounds generally exhibit higher affinity for SSTR2 [36].

## 3. Radiolabeled Somatostatin Analogs

A radiolabeled SSTA consists of three main components: a receptor-binding peptide, a radionuclide, and a chelator that links the two. Commonly used chelators include DOTA (tetraazacyclododecane tetraacetic acid) and DTPA (diethylenetriaminepentaacetic acid), while the radionuclide is often a radiometal [5]. Following radiolabeling, it is important that the affinity of the peptide for SSTRs, particularly SSTR2, is preserved.

Several radiolabeled SSTAs have been developed and evaluated both preclinically and clinically for diagnostic imaging and therapeutic purposes, primarily in patients with SSTR-expressing NETs. After systemic administration, radiolabeled SSTAs bind to SSTRs expressed on the tumor cell surface. The receptor-ligand complex is subsequently internalized through receptor-mediated endocytosis, resulting in intracellular retention of the radionuclide, primarily within lysosomes, thereby increasing retention of radioactivity in tumor tissue [2,5].

The radiolabeled SSTA [^177^Lu]Lu-DOTATATE (^177^Lu-oxodotreotide, ^177^Lu-octreotate) has been used extensively in preclinical NET models and is today routinely used for treatment of patients with disseminated tumor disease [5,38,39,40,41]. Another clinically established radiopharmaceutical is [^177^Lu]Lu-DOTATOC (^177^Lu-edotreotide, ^177^Lu-octreotide), which has demonstrated efficacy in patients with advanced NETs and is currently being evaluated in several phase III clinical trials [18]. Both compounds target SSTR2, although DOTATATE exhibits higher affinity for SSTR2 and has therefore become the most widely used peptide for PRRT [36,37]. ^177^Lu has suitable physical properties, with high emission of β-particles of relatively low energy (approximately 2 mm maximum tissue penetration), resulting in a higher absorbed fraction in tumors and lower renal toxicity compared with [^90^Y]Y-DOTATATE [10]. Additionally, ^177^Lu emits γ-photons useful for scintigraphy, enabling dosimetric evaluation and post-treatment follow-up [8].

[Lu]Lu-DOTATATE (trademarked Lutathera^®^) was approved by both the EMA and FDA in 2018 for treatment of patients with advanced SSTR-positive gastroenteropancreatic NETs. The currently approved treatment protocol consists of four administrations of 7.4 GBq separated by approximately 8 weeks (which may be extended if toxicity occurs), together with concomitant administration of amino acids for renal protection and long-acting (LA) SSTAs [11,12].

## 4. Clinical Experience of Therapy with ^177^Lu-Labeled SSTAs

### 4.1. Clinical Experience of [^177^Lu]Lu-DOTATATE Therapy

[^177^Lu]Lu-DOTATATE has been used clinically since the early 2000s, primarily for treatment of patients with GEP-NETs. Overall, the clinical outcome has been relatively successful, with prolonged PFS and OS, although complete responses and cures remain uncommon [14]. The first clinical studies demonstrated encouraging tumor responses, prolonged disease stabilization, and acceptable toxicity profiles [42,43]. Subsequent prospective and retrospective studies consistently confirmed objective response rates of approximately 20–40%, disease control rates exceeding 70% in many cohorts, and median PFS commonly ranging between 20 and 40 months [11,42,43,44,45,46,47,48,49,50,51,52,53,54] (Table 1). Clinical benefit has also been demonstrated in specific patient populations, including patients with functioning pancreatic NETs, paragangliomas, pheochromocytomas, heavily pretreated pancreatic NETs, and lung NETs [55,56,57,58,59].

Across studies, a substantial proportion of patients achieve partial response or durable stable disease. The NETTER-1 phase III clinical study, involving patients with advanced midgut NETs, showed initially promising results: after 20 months the progression-free survival was 65% in patients who received [^177^Lu]Lu-DOTATATE together with octreotide long-acting repeatable (LAR), compared with 11% in the control group receiving octreotide LAR alone [7,12,13]. However, at long-term follow-up from the same study (after a median follow-up of 76 months), the difference in overall survival was not statistically significant [13]. The phase III NETTER-2 study (NCT03972488) evaluated [^177^Lu]Lu-DOTATATE as first-line treatment in patients with newly diagnosed advanced grade 2 or grade 3 well-differentiated SSTR-positive GEP-NETs. Patients received [^177^Lu]Lu-DOTATATE in combination with octreotide LAR and were compared with a control group receiving octreotide LAR alone [70]. Preliminary results demonstrated a median progression-free survival of 23 months in the [^177^Lu]Lu-DOTATATE group compared with 8 months in the control group, supporting the role of PRRT in earlier treatment lines. Additional randomized evidence has recently been reported for pancreatic NETs. In the phase II OCLURANDOM trial, patients with pretreated, progressive, SSTR-positive metastatic pancreatic NETs were randomized to [^177^Lu]Lu-DOTATATE or sunitinib. The 12-month progression-free survival rate was 80.5% with [^177^Lu]Lu-DOTATATE and 41.9% with sunitinib, with fewer grade 3–4 adverse events in the PRRT group [69].

Clinical experience accumulated over more than two decades indicates that [^177^Lu]Lu-DOTATATE therapy is generally well tolerated and that kidneys and bone marrow are the principal dose-limiting organs (Table 1) [9,16,43]. Nausea and vomiting are common acute, but usually mild, side effects and are primarily related to co-administration of positively charged amino acids used to reduce renal uptake and retention of ^177^Lu and thereby decrease the risk of late nephrotoxicity [9,51]. Bone marrow suppression is a subacute side effect that typically occurs 4–6 weeks after treatment and may manifest as reductions in hemoglobin levels, platelet counts, and/or white blood cell counts [4,9].

Hematological toxicity of grade 3 or higher has been reported in a minority of patients across studies [43,47,52]. While severe long-term hematological complications such as leukemia or myelodysplastic syndrome (MDS) occur in approximately 1–2% of treated patients [9,11,43]. Long-term follow-up studies and dosimetry-guided treatment protocols have generally demonstrated acceptable renal safety and favorable long-term tolerability, particularly when amino acid nephroprotection is used and absorbed dose constraints are respected [48,54]. These findings support the favorable therapeutic index of [^177^Lu]Lu-DOTATATE and have contributed to its establishment as a standard treatment for patients with advanced SSTR-positive NETs.

### 4.2. Clinical Experience of Therapy with Other ^177^Lu-Labeled SSTAs

New ^177^Lu-labeled SSTAs are continuously being developed with the aim of improving tumor uptake, prolonging tumor retention, increasing absorbed dose to tumor tissue, or reducing uptake in normal organs. Although [^177^Lu]Lu-DOTATATE remains the most widely used radiopharmaceutical for PRRT, several alternative agonists and antagonists have demonstrated encouraging preclinical and clinical results.

One clinically established alternative is [^177^Lu]Lu-DOTATOC, which differs from DOTATATE in receptor affinity profile and exhibits somewhat lower affinity for SSTR2. Nevertheless, clinical studies have demonstrated favorable efficacy and safety in patients with advanced NETs, with objective response rates and progression-free survival comparable to those reported for [^177^Lu]Lu-DOTATATE in select patient populations [18]. The phase III COMPETE trial compared [^177^Lu]Lu-DOTATOC with everolimus in patients with progressive grade 1–2 GEP-NETs [71]. The study demonstrated significantly prolonged progression-free survival, higher objective response rates, and fewer grade 3–4 treatment-related adverse events in patients treated with [^177^Lu]Lu-DOTATOC, providing the first phase III evidence establishing the superiority of DOTATOC-based PRRT over targeted therapy with everolimus. These results contributed to the FDA acceptance of a new drug application for [^177^Lu]Lu-DOTATOC (under nonproprietary name [^177^Lu]Lu-edotreotide (ITM-11)) for treatment of GEP-NETs [72].

An LA SSTA using Evans blue (EB) as an additional linker (EB-TATE) was developed to prolong circulation through reversible albumin binding [24]. Clinical evaluation of [^177^Lu]Lu-DOTA-EB-TATE demonstrated higher tumor uptake, prolonged retention, and increased tumor absorbed dose compared with [^177^Lu]Lu-DOTATATE, but uptake and retention in normal tissues were also increased [25]. In an ongoing phase I study (NCT03478358), higher administered activities were associated with increased hematological and hepatic toxicity, while subsequent evaluation of multiple treatment cycles suggested improved tumor control at higher activities but treatment-limiting toxicity in some patients [23,73]. Further modification of the albumin-binding approach has led to the development of [^177^Lu]Lu-LNC1010, an optimized long-acting SSTR2-targeting radiopharmaceutical designed to improve stability and pharmacokinetics compared with EB-TATE. Preclinical studies demonstrated increased tumor uptake and prolonged retention in AR42J xenografts, while initial clinical evaluation in patients with advanced NETs demonstrated high tumor absorbed doses and encouraging preliminary therapeutic responses [74].

Another approach is to target SSTRs using receptor antagonists rather than agonists. In a first-in-human study, the SSTR antagonist [^177^Lu]Lu-DOTA-LM3 demonstrated higher tumor uptake than [^177^Lu]Lu-DOTATATE in patients with metastatic NETs, albeit at the expense of increased uptake in several normal tissues, including kidneys and liver [19].

Collectively, these studies demonstrate that modifications of peptide structure, pharmacokinetics, and receptor-binding characteristics can substantially alter tumor and normal-organ dosimetry. While [^177^Lu]Lu-DOTATATE remains the current standard of care, newer agonists and antagonists have the potential to improve PRRT efficacy and broaden future treatment options for patients with SSTR-positive NETs.

### 4.3. Ongoing Clinical Studies

Several ongoing clinical studies are expected to define the future role of PRRT in NETs and other SSTR-positive malignancies. Recent reviews and large clinical series confirm that PRRT has become an established treatment modality for advanced NETs, while also highlighting several remaining questions regarding optimal patient selection, treatment sequencing, dosimetry, retreatment, and the development of novel radiopharmaceuticals (Figure 1) [26,30,33].

The NETTER-3 phase III study (NCT06784752) is evaluating [^177^Lu]Lu-DOTATATE in patients with advanced grade 1–2 GEP-NETs. This study broadens the clinical development program beyond the populations evaluated in NETTER-1 and NETTER-2 and may clarify the role of PRRT earlier in the disease course. In parallel, the Italian multicenter observational REAL-LU study (NCT04727723) is evaluating long-term effectiveness, safety, and treatment patterns of [^177^Lu]Lu-DOTATATE in routine clinical practice [31]. Complementing these prospective studies, a recent retrospective analysis of 173 patients evaluated dose delays and dose reductions during routine [^177^Lu]Lu-DOTATATE therapy. The study demonstrated that treatment modifications are relatively common in clinical practice, primarily because of hematological toxicity or intercurrent clinical factors, highlighting the importance of individualized treatment scheduling while maintaining favorable overall tolerability [75].

Alternative radiolabeled SSTAs are also being investigated. The phase III COMPOSE study (NCT04919226) is currently comparing [^177^Lu]Lu-DOTATOC with standard systemic therapy in patients with aggressive grade 2–3 GEP-NETs [21]. Additional studies are exploring the use of PRRT in earlier treatment lines and alternative treatment sequences (NCT05064150, NCT05894486). Recent reviews suggest that the results of these studies may substantially influence future treatment algorithms and the positioning of PRRT within NET management.

Personalized dosimetry-guided treatment approaches are under investigation (NCT05387603, NCT04903899, NCT02743741, NCT06395402, NCT06256705, NCT04917484, NCT03454763), reflecting increasing interest in individualized activity administration rather than fixed treatment schedules. Recent clinical dosimetry data demonstrated that tumor absorbed dose may decrease over successive treatment cycles, whereas kidney absorbed dose remained relatively stable, resulting in declining tumor-to-kidney absorbed dose ratios during therapy [76]. Cumulative renal absorbed dose was also associated with subsequent decline in renal function. The potential role of individualized dosimetry in treatment optimization is further discussed in Section 5.2. Other studies are evaluating retreatment strategies following prior radionuclide therapy (NCT04954820, NCT05773274, NCT01876771), an increasingly important clinical question as more patients receive PRRT earlier in the disease course.

SSTR antagonists are also undergoing further clinical evaluation, including satoreotide-based radiopharmaceuticals in patients with meningioma (NCT04997317), while [^177^Lu]Lu-DOTA-LM3 and related compounds continue to be investigated as alternatives to agonist-based PRRT [19].

Beyond conventional β-emitting radionuclides, α-particle therapy is attracting increasing interest [20]. Ongoing studies are evaluating alpha-emitting SSTR-targeted radiopharmaceuticals (NCT05477576, NCT05636618, NCT05153772, NCT06427798). Initial dosimetry results from the phase 1b component of ACTION-1 further demonstrated the feasibility of image-based [^225^Ac]Ac-DOTATATE dosimetry and showed favorable tumor-to-normal-tissue absorbed-dose ratios [77]. Because α-particles deposit substantially higher linear energy transfer over a shorter path length than β-particles, these agents may offer advantages in resistant disease and micrometastatic tumor deposits while potentially reducing irradiation of surrounding normal tissues. Although clinical experience remains limited, alpha therapy is widely considered one of the most actively investigated next-generation developments in radionuclide therapy. A recent retrospective comparative study evaluating salvage [^225^Ac]Ac-DOTATATE versus repeat [^177^Lu]Lu-DOTATATE in patients progressing after an initial response to PRRT suggested improved disease control following α-emitter therapy, supporting the potential role of targeted α therapy for patients with acquired resistance to β-emitting radionuclides [68]. However, prospective randomized studies are still lacking. Recent studies have highlighted α-emitter PRRT as a potential strategy for overcoming resistance to conventional β-emitting radiopharmaceuticals and for improving responses in patients with advanced disease [17,20,22]. In addition to emerging α-emitting radiopharmaceuticals, alternative β- and electron-emitting radionuclides, including ^90^Y, ^161^Tb, and ^153^Sm, are being used or investigated for SSTR-targeted radionuclide therapy and provide different physical characteristics that may offer opportunities for further treatment optimization [78,79,80,81,82].

Ongoing studies are evaluating SSTR-targeted radionuclide therapy in other neuroendocrine and non-neuroendocrine malignancies, reflecting increasing interest in increasing the clinical applications of theranostics beyond conventional GEP-NET indications. (NCT04529044, NCT06045260, NCT06991738, NCT03971461, NCT05918302, NCT04949282, NCT05691465, NCT07538791, NCT04665739, NCT06121271). Furthermore, PRRT is being explored in pediatric and adolescent patients with SSTR-positive tumors, reflecting the gradual expansion of radionuclide therapy beyond traditional adult NET populations (NCT06607692, NCT06441331).

In parallel with the development of new treatment strategies, increasing attention is being directed toward optimization of response assessment following PRRT. Conventional radiographic assessment using RECIST 1.1 may underestimate treatment response in slowly growing NETs, where changes in tumor size often occur late despite substantial biological response. Satapathy et al. proposed the Response Evaluation Criteria in Neuroendocrine Tumors (RECIN), an SSTR PET/CT-based framework incorporating changes in summed SULpeak following [^177^Lu]Lu-DOTATATE therapy [83]. In a post hoc analysis of the phase II LuCAP trial, RECIN identified a substantially larger proportion of responding patients than RECIST and demonstrated improved prediction of progression-free survival [61,83]. The ENETS SSTR-PET response assessment framework (SSTR-PeRForm) emphasizes the need for standardized PET-based response evaluation in patients treated with PRRT [84]. Although these approaches require prospective validation, they illustrate the growing recognition that molecular imaging may complement or improve conventional anatomical response assessment in NETs.

Taken together, current clinical development is moving in several directions, including expansion of PRRT to new patient populations, optimization of treatment delivery through personalized dosimetry, evaluation of retreatment strategies, development of novel agonists and antagonists, and introduction of alternative therapeutic radionuclides. Results from these studies are expected to define the future role of PRRT and may lead to substantial changes in radionuclide therapy protocols over the coming years. Recent reviews suggest that the next decade of PRRT development will likely be characterized by increasing personalization, broader clinical indications, and the introduction of new radiopharmaceutical platforms beyond conventional [^177^Lu]Lu-DOTATATE.

## 5. Strategies to Improve Therapy with ^177^Lu-Labeled SSTAs

Several strategies to optimize treatment of NETs have been suggested, including treatment individualization, methods to increase the effect on tumor tissue, and methods to reduce toxicity in normal tissues [34,85,86]. The therapeutic effect of PRRT depends on several consecutive and interconnected steps, including SSTR expression and availability, radiopharmaceutical binding and tumor uptake, retention and absorbed dose, induction of radiation damage, and the subsequent balance between cellular repair and stress responses and tumor cell death. Treatment may therefore be enhanced at several points in this process (Figure 1). These include increasing receptor availability and tumor targeting, improving radiopharmaceutical delivery and tumor dosimetry, preventing repair of radiation-induced damage, suppressing cellular stress adaptation and survival, and promoting tumor cell elimination [34]. The strategies discussed below act at one or several of these steps and are supported by different levels of experimental and clinical evidence. Importantly, increased SSTR expression does not necessarily result in increased radiopharmaceutical uptake, and increased tumor uptake does not necessarily translate into an improved therapeutic effect. Similarly, strategies that increase the radiation response in tumor cells may also affect normal tissues. The therapeutic effect must therefore be considered in relation to both tumor response and toxicity in dose-limiting normal tissues. Strategies that interfere with DNA repair, apoptosis regulation, or cellular stress responses may also increase radiation effects in normal tissues and thereby narrow the therapeutic window [87,88,89,90]. The therapeutic window of combination treatment may also depend on patient-related factors such as age, comorbidities, prior treatments, and baseline organ function [91,92,93]. This may become increasingly relevant as PRRT and combination strategies are evaluated in broader patient populations, including children and adolescents [94,95,96,97,98,99,100]. Recent population pharmacokinetic and dosimetry analyses of [^177^Lu]Lu-DOTATATE in adolescents demonstrated exposure and kidney and bone marrow dosimetry broadly comparable to those in adults, supporting the feasibility of the established adult dosing regimen in adolescent patients [101].

Below, results from preclinical and clinical studies addressing these different approaches are summarized (Table 2, Table 3, Table 4 and Table 5). To facilitate understanding, a summary of cell types and cell lines mentioned is given in Table 6, while the principal treatment-enhancement strategies and their level of evidence are summarized in Table 7. It should be noted that not all cell lines historically used as neuroendocrine tumor models are considered authentic NET cell lines. KRJ-I, L-STS and H-STS were subsequently shown to be lymphoblastoid rather than neuroendocrine in origin and therefore do not represent bona fide NET cell lines. Nevertheless, H-STS and KRJ-I have been reported to retain aspects of GEP-NET regulatory networks and drug-response profiles and have therefore continued to be used in selected mechanistic and drug-screening studies. Findings obtained using these models should be interpreted with appropriate caution [102,103].

### 5.1. Increasing Tumor Targeting and Receptor Availability

SSTR expression and availability are important determinants of tumor uptake and the efficacy of SSTR-targeted PRRT [3]. However, when high amounts of radiolabeled SSTAs are administered, saturation of SSTRs may occur, resulting in reduced binding and uptake per administered activity and consequently lower absorbed dose to the tumor and a lower tumor-to-organ absorbed dose ratios [159]. Strategies aimed at increasing receptor expression or availability may therefore increase tumor targeting (Figure 1). Several approaches have been explored in vitro and in vivo in animal models, and to a limited extent in patients, including pre-stimulation with radiation, modulation by unlabeled SSTAs, hormones, epigenetic-modifying drugs, and other pharmacological agents (Table 2, Table 3, Table 4 and Table 5) [34]. Importantly, increased SSTR expression does not necessarily result in increased tumor uptake or improved therapeutic efficacy, since these also depend on factors such as receptor saturation, radiopharmaceutical pharmacokinetics and retention, and radiation sensitivity of the tumor [160]. SSTR PET/CT may also provide a means of identifying patients with low or heterogeneous tumor SSTR expression who could potentially benefit from strategies aimed at increasing receptor availability, although the clinical value of such an approach remains to be established.

#### 5.1.1. Radiation-Induced Modulation of SSTR Expression

One approach to increase SSTR expression and receptor availability is exposure to ionizing radiation. This phenomenon has been studied both in vitro and in animal models, and is summarized as a potential strategy for increasing tumor targeting in Table 7. Several studies suggest that radiation-induced receptor modulation may increase subsequent uptake of radiolabeled SSTAs, although the effect depends on absorbed dose and timing and may also involve reduced receptor saturation.

An in vitro study on human small cell lung cancer H69 cells demonstrated a twofold higher expression of SSTR2 mRNA 3 days after external beam radiation (EBR) exposure 2–8 Gy with 100 keV (max) X-rays, resulting in 1.5–3 times higher uptake of [^177^Lu]Lu-DOTATATE in the cells [161]. In a similar study, when the H69 cells were irradiated to lower absorbed doses (0.12–6.0 Gy) with 100 keV (max) X-rays, the *SSTR1*, *SSTR2* and *SSTR5* mRNA expression increased 1.5–2 times at the higher absorbed doses, and the binding of [^177^Lu]Lu-DOTATATE was 2–3 times higher for almost all absorbed doses compared with nonirradiated cells [162].

Increased uptake after prior irradiation was also demonstrated in vivo in the GOT1 human siNET xenograft model, where injection with a low amount of [^177^Lu]Lu-DOTATATE 3–13 days prior to injection of another radiolabeled SSTA, [^111^In]In-DOTATOC, caused up to twofold higher activity concentration of ^111^In compared with ^177^Lu in the tumors [163].

In two studies on rats with rat pancreatic CA20948 tumors, exposure to either [^177^Lu]Lu-DOTATATE or [^111^In]In-DOTATOC initially resulted in lower expression of SSTR2, but in regrowing tumors the SSTR2 expression was 2–5 times higher, indicating a radiation-induced effect [164,165]. These results suggest that SSTR regulation may be time-dependent, with early receptor downregulation followed by increased expression during tumor regrowth.

Altogether, the available evidence suggests that ionizing radiation can modulate SSTR expression and subsequent radiolabeled SSTA uptake, although the effects depend on absorbed dose and timing. The therapeutic effects of fractionated administration and its influence on tumor and normal-tissue absorbed dose are discussed in Section 5.2.

#### 5.1.2. SSTR Modulation by SSTAs

Exposure to SSTAs may result in down- or upregulation or saturation of SSTRs in vitro and in vivo, dependent on the time and dose of SSTAs given. These effects are relevant for treatment optimization strategies listed in Table 7, where SSTA priming is included as a potential approach to modulate receptor availability.

In vitro data on AR42J cells showed that a short incubation with high amounts of TOC resulted in downregulation of SSTR2, and the level was restored after 24 h and required de novo SSTR2 synthesis [166]. The downregulation was not seen with low amounts of TOC. The same study showed similar downregulation in vivo after a single injection of TOC in SCID mice transplanted with AR42J cells, while continuous exposure to low amounts of TOC resulted in upregulation of SSTR2 after 7 days. Recent in vitro studies also demonstrated that unlabeled DOTATATE can promote release of previously internalized [^177^Lu]Lu-DOTATATE, further indicating that the timing and nature of SSTR ligand exposure may influence radiopharmaceutical retention [160].

In CA20948 tumor-bearing rats, acute injection with very high amounts of TOC (175 µg/kg) resulted in reduced uptake of ^68^Ga-DOTATATE if given simultaneously, while no effects were found after 4 h–1 d, or when given 20 min prior to ^68^Ga-DOTATATE, indicating a competition for receptor binding [167]. In male Wistar rats, priming with TOC 0, 10 or 20 min prior to injection of [^111^In]In-DOTATOC decreased the uptake in SSTR-expressing normal tissues (adrenals and pancreas), but not when given 30 min after [^111^In]In-DOTATOC injection [168]. However, no data on tumor tissue was available, and there may be differences in receptor expression and kinetics between tumor and different normal tissues.

In patients with metastatic NETs undergoing treatment with LA SSTAs, pretreatment with lanreotide 24 h in advance resulted in higher tumor uptake of [^68^Ga]Ga-DOTATATE, while uptake in several normal tissues was somewhat reduced [169]. In another study comparing patients receiving different LA SSTA regimens, no clear effect on tumor uptake of [^68^Ga]Ga-DOTATATE was observed between groups, while liver uptake was reduced after initiation of LA SSTA treatment [170]. A longitudinal PET study evaluating serial [^68^Ga]Ga-DOTATOC imaging during PRRT demonstrated dynamic changes in physiologic SSTR expression in normal organs over the course of treatment. The study also highlighted that different LA SSTA regimens may influence normal-organ tracer uptake and suggested that serial SSTR PET imaging may provide insight into treatment response and organ dosimetry during PRRT [171].

Overall, SSTA-induced modulation of SSTR expression appears to depend strongly on dose, timing, and treatment schedule. Available data suggest that high-dose acute exposure may lead to receptor saturation or transient downregulation, whereas lower or prolonged exposure may result in receptor upregulation. Together, these findings support careful scheduling of SSTA administration in relation to radiolabeled SSTA therapy, as summarized in Table 7, although clinical evidence for consistent improvement in therapeutic efficacy remains limited.

#### 5.1.3. SSTR Modulation by Other Hormones

Multiple studies demonstrate that different hormones may lead to modulation of SSTR expression, mainly in preclinical models, as summarized in Table 7. In addition, interactions between SSTR signaling and endocrine pathways have been described in physiological and tumor contexts, suggesting that hormonal regulation may influence receptor expression and function [35,36,172].

In a study on hypothyroid mice bearing murine TtT-97 thyrotropic tumors, administration of thyroid hormone (T4) resulted in upregulation of *SSTR1* and *SSTR5* mRNA and increased cell surface expression of high affinity SSTRs [173].

Exposure of AR42J cells to epidermal growth factor (EGF) and gastrin showed increased *SSTR2* mRNA expression, while expression of *SSTR1* and *SSTR3* was reduced, with no alteration in receptor affinity [174].

Studies on the effect of estrogen and the estrogen receptor antagonist tamoxifen on breast cancer cell lines (ZR-75-1 and T47D) demonstrated that tamoxifen increased *SSTR1* mRNA levels, while *SSTR2* expression was upregulated after treatment with either tamoxifen or estradiol [175].

Collectively, hormonal regulation can influence SSTR expression, although the effects appear to be receptor subtype-specific and dependent on the signaling context. It should be noted that these studies are limited to in vitro and animal models, and no clinical data currently demonstrate hormone-induced SSTR upregulation in the context of PRRT (Table 7).

#### 5.1.4. Epigenetic Modulation of SSTR Expression and Tumor Targeting

The role of epigenetics has received increasing attention in cancer research, and several epigenetic modifiers have been investigated in NETs. Modification of the epigenome, including DNA methylation (e.g., hypermethylation of CpG islands in promoter regions) and chromatin remodeling (e.g., histone modifications) are well-known mechanisms involved in regulation of gene expression [176,177]. Epigenetic alterations may lead to silencing of tumor suppressor genes or activation of oncogenes. SSTR expression may be increased by epigenetic-modifying drugs (epidrugs), such as DNA methyltransferase inhibitors (DNMTis) and histone deacetylase inhibitors (HDACis), as summarized in Table 7.

Preclinical studies of DNMTis and HDACis have demonstrated upregulation of *SSTR2* expression. Methylation at the upstream promoter region of *SSTR2* has been shown to be inversely associated with mRNA expression levels in several cell lines, while epidrug treatment resulted in a significant increase in *SSTR2* expression [178]. Another study on human pancreatic NET cell lines BON and QGP-1 showed increased *SSTR2* mRNA expression and increased uptake of [^125^I]I-octreotide after treatment with the epidrugs 5-aza-2′-deoxycytidine (5-aza-dC) and HDACi valproic acid (VPA) [179]. When one low (QGP-1), and two high (BON and CM) methylated pNET cells were incubated with the DNMTi guadecitabine, SSTR2 expression was increased, demonstrated by higher binding of the radiolabeled SSTA ^18^F-FET-bAG-TOCA ([^18^F]F-FETO) [180]. Corresponding in vivo studies in nude mice bearing QGP-1 and BON tumors showed higher uptake of [^18^F]F-FETO in BON, but not in QGP-1 tumors after treatment with guadecitabine. However, in a therapeutic experiment in mice with BON tumors, treatment with [^177^Lu]Lu-DOTATATE in combination with guadecitabine did not result in an improved therapeutic effect despite increased receptor expression [180].

Incubation with HDAC inhibitors such as thailandepsin A (TDP-A), romidepsin (FK228), suberoylanilide hydroxamic acid (SAHA), AB3, and VPA resulted in increased SSTR2 mRNA and protein expression in several NET cell lines, including TT and MZ-CRC-1 (medullary thyroid carcinoma), BON, and H727 (pulmonary carcinoid), with the most pronounced effect observed in H727 [181,182]. The functionality of SSTR2 was confirmed by increased uptake of [^68^Ga]Ga-DOTATATE after HDACi exposure [181,182]. In a subsequent study, increased gene expression of SSTR2 was observed in both BON and QGP-1 cells after exposure to these HDAC inhibitors, although increased protein expression was primarily seen in BON cells [182]. However, increased expression of SSTR2 protein was only obtained in BON cells with lower SSTR2 expression than QGP-1 cells after exposure to TDP-A, FK228, SAHA, VPA, and AB3. In vivo, mice bearing BON tumors showed increased uptake of [^68^Ga]Ga-DOTATATE after pretreatment with FK228 [182]. Auernhammer et al. expanded this concept by evaluating combined epigenetic modulation using the class I HDAC inhibitor entinostat together with the LSD1 inhibitor CC-90011 [183]. Combined treatment markedly increased SSTR2 expression and enhanced uptake of [^18^F]SiTATE in BON, H727 and QGP-1 cells compared with either treatment alone, suggesting that simultaneous targeting of multiple epigenetic regulatory pathways may provide a more effective strategy for increasing SSTR expression than HDAC inhibition alone. Although reports remain limited to in vitro models, they support epigenetic modulation as a candidate strategy for improving molecular imaging and potentially PRRT in tumors with low baseline SSTR2 expression. In vitro studies on three NET cell lines (GOT1, KRJ-I, and BON) demonstrated increased SSTR2 expression after incubation with VPA [184]. Similarly, immunohistochemical staining showed increased expression of membrane-bound SSTR2 in HEKsst2 cells after exposure to 5-aza-dC and VPA, accompanied by increased [^177^Lu]Lu-DOTATATE uptake and therapeutic effect [141].

Klomp et al. highlighted the complexity of translating HDACi-induced SSTR2 upregulation into improved PRRT efficacy in H69 cells. VPA increased SSTR2 expression in vitro and enhanced tumor uptake of [^177^Lu]Lu-DOTATATE in vivo; however, the increased uptake appeared to be related primarily to altered pharmacokinetics and prolonged radiotracer circulation rather than increased receptor expression in tumor tissue [185]. Increased renal tubular damage was observed, suggesting that epigenetic priming strategies may also influence the therapeutic window of PRRT. In a subsequent study, Klomp et al. screened several HDAC inhibitors for their ability to increase SSTR2 expression and radiolabeled DOTATATE uptake. Although robust increases in receptor expression were observed in vitro, corresponding increases in radiotracer uptake in vivo were more limited, emphasizing the challenges associated with translating epigenetic receptor modulation into improved PRRT efficacy [186].

Similarly, Sharma et al. demonstrated that pharmacological upregulation of SSTR2 in receptor-deficient pancreatic neuroendocrine tumor models resulted in increased uptake of [^177^Lu]Lu-DOTATATE and improved therapeutic efficacy [187]. This provides further support for the concept that increasing SSTR expression can enhance PRRT responses, particularly in tumors with low baseline receptor expression, although the relative contributions of receptor upregulation, altered pharmacokinetics, and radiosensitization remain incompletely understood.

Clinical studies investigating epigenetic priming to improve imaging and therapy of NETs have been initiated. The LANTana phase Ib study is evaluating whether treatment with the DNMTi ASTX727 (decitabine + cedazuridine) can increase SSTR2 expression (assessed by [^68^Ga]Ga-DOTATATE PET) in NET patients with low baseline uptake, with the aim of enabling subsequent treatment with [^177^Lu]Lu-DOTATATE [188].

In summary, epigenetic-modifying drugs consistently increase SSTR expression in several preclinical models, and increased radiotracer uptake has also been demonstrated. However, increased receptor expression does not consistently translate into increased tumor uptake or improved therapeutic efficacy, and altered pharmacokinetics and normal-tissue exposure may also influence treatment outcome. Clinical evidence remains limited, although ongoing studies are evaluating epigenetic priming in patients with low baseline SSTR expression (Table 7) [189].

#### 5.1.5. SSTR Modulation by Other Pharmacological Agents

Several chemotherapy drugs have been shown to modulate SSTR expression, mainly in preclinical models, as summarized in Table 7. These effects are often time- and dose-dependent and may involve both direct regulation of receptor expression and indirect effects on cellular stress and differentiation pathways.

In vitro studies on BON and H727 cells showed that incubation with chemotherapy drugs such as temozolomide resulted in higher SSTR2 mRNA expression after 3–7 days, accompanied by increased uptake of [^177^Lu]Lu-DOTATATE and decreased cell proliferation rate [190].

Gemcitabine is a nucleoside analog of deoxycytidine that inhibits DNA synthesis and is clinically used to treat several types of cancer, including pancreatic and lung cancer. Pretreatment of Capan-2, AR42J and H69 cells with gemcitabine initially caused downregulation, but after 4 days resulted in upregulation of *SSTR* expression, with increased binding and uptake of [^177^Lu]In-DOTATOC [191].

Glucocorticoids (GCs) may induce downregulation of *SSTR2* expression. However, the GC receptor antagonist relacorilant inhibited GC-mediated suppression of SSTR2 and increased SSTR2 expression in vitro in the murine At-T20 cell line (pituitary cancer) [192]. High concentrations of relacorilant also increased *SSTR2* mRNA expression above basal levels. In a patient with ACTH-secreting bronchial NET, no uptake of ^68^Ga-labeled SSTA was observed in the pituitary gland at baseline, whereas treatment with relacorilant restored *SSTR2* expression and tracer uptake [193].

Similar effects of chemotherapy-induced modulation of receptor expression and radiosensitivity have been described in other tumor models, although direct evidence linking these effects to improved PRRT efficacy remains limited [194,195].

Overall, chemotherapy-induced modulation of SSTR expression appears to vary according to treatment schedule and tumor type. Although several agents have increased receptor expression and radiotracer uptake in preclinical models, clinical evidence supporting improved therapeutic efficacy in combination with [^177^Lu]Lu-DOTATATE remains limited (Table 2, Table 5 and Table 7).

### 5.2. Improving Tumor Radiopharmaceutical Uptake and Absorbed Dose

In addition to increasing SSTR expression or receptor availability, the therapeutic effect of PRRT may be improved by increasing the amount and retention of radioactivity in tumor tissue and thereby increasing tumor absorbed dose. This may be achieved through modification of the radiopharmaceutical, treatment schedule, or administered activity. Importantly, increased tumor uptake or absorbed dose is only beneficial if the corresponding exposure of dose-limiting normal tissues remains acceptable. Several approaches targeting this step of the therapeutic process are currently being investigated, including fractionated administration, modification of radiopharmaceutical pharmacokinetics, use of SSTR antagonists, and individualized dosimetry (Figure 1, Table 7).

Fractionation of radiolabeled SSTAs may increase tumor radiation delivery through educed receptor saturation and repeated targeting of available receptors. In the GOT1 animal model, a priming/fractionation schedule using a first injection with low activity of [^177^Lu]Lu-DOTATATE followed by a second injection with higher activity 24 h late resulted in almost twofold higher mean absorbed dose to the tumor and lower absorbed dose to the kidneys compared with administration of the same total activity at one time point [196]. In the same model, therapeutic effects were evaluated after 1–3 injections with higher amounts of [^177^Lu]Lu-DOTATATE given 24 h, 1 week, or 2 weeks apart [197]. Fractionation resulted in higher tumor volume reduction, delayed tumor regrowth, and prolonged overall survival. Higher tumor volume reduction was also reported in an H69 small cell lung cancer mouse model after [^177^Lu]Lu-DOTATATE fractionation [198], while fractionation of [^177^Lu]Lu-DOTATATE or [^67^Cu]Cu-Sar-octreotate resulted in higher therapeutic effects in mice carrying AR42J tumors [199]. In a case study of a patient with siNET, higher tumor uptake of [^111^In]In-DOTATOC was obtained when the radiopharmaceutical was administered as two fractions 24 h apart compared with one administration [200]. Together, these studies indicate that fractionation can influence radiopharmaceutical uptake, tumor and normal-organ absorbed dose, and therapeutic efficacy. However, most evidence is preclinical and the optimal fractionation schedule and clinical benefit remain to be established.

Modification of radiopharmaceutical pharmacokinetics represents another approach to increase tumor radiation delivery. Albumin-binding compounds such as EB-TATE and the more recently developed LNC1010 prolong circulation and can thereby increase tumor uptake, retention, and absorbed dose [24,74]. However, as described in Section 4.2, prolonged circulation may also increase normal-tissue exposure and does not necessarily improve the therapeutic ratio.

SSTR antagonists provide another means of increasing tumor targeting. Despite limited or absent receptor internalization, antagonists can bind to a larger number of receptor sites and may therefore achieve high tumor uptake and retention. Clinical studies have demonstrated increased tumor uptake, although normal-tissue uptake may also increase, as discussed in Section 4.2 [19].

Finally, individualized dosimetry provides a means to adapt the administered activity according to patient-specific tumor and normal-organ absorbed doses rather than using a fixed treatment schedule. This approach may allow treatment intensity to be adapted to normal-tissue tolerance while aiming to increase tumor absorbed dose. Recent clinical studies have further demonstrated associations between tumor absorbed dose and treatment outcome. Macsuka et al. found that both cumulative tumor absorbed dose and changes in absorbed dose per administered activity over successive treatment cycles were associated with radiological response to [^177^Lu]Lu-DOTATATE [201]. Similarly, higher mean tumor absorbed dose was independently associated with improved progression-free and overall survival in patients with grade 1–2 NETs treated with individualized [^177^Lu]Lu-DOTATATE [202]. However, whether individualized dosimetry results in improved clinical outcomes compared with fixed-activity treatment remains under investigation [27,28,30,32,76]. Preclinical studies further demonstrate the importance of considering absorbed dose together with biological response. Using image-based cellular dosimetry in GOT1 and NCI-H69 models, Tamborino et al. established dose–effect relationships for [^177^Lu]Lu-DOTATATE and demonstrated differences between PRRT and EBR as well as between two- and three-dimensional tumor models [203]. These findings illustrate that absorbed dose alone does not fully describe the biological response and support integration of dosimetry and radiobiology when evaluating PRRT optimization strategies.

### 5.3. Preventing Repair of Radiation-Induced Damage

Ionizing radiation induces cellular damage through direct ionization of biomolecules and indirectly through generation of reactive oxygen species (ROS), resulting in several forms of DNA damage [204,205]. Among these, DNA double-strand breaks are considered particularly important for radiation-induced cell death [206]. However, tumor cells can activate DNA damage response (DDR) pathways that detect and repair radiation-induced DNA lesions, thereby limiting the therapeutic effect. Inhibition of DNA repair therefore represents a strategy to increase tumor cell sensitivity to [^177^Lu]Lu-DOTATATE [207]. Several components of the DDR have been investigated as potential targets for combination therapy, with PARP and DNA-PK inhibitors currently providing the most direct preclinical and early clinical evidence in combination with PRRT. A recent direct comparison of PRRT and EBR in NET cell models demonstrated overlapping transcriptional responses dominated by DNA damage response and cell-cycle pathways, although the response to PRRT was delayed. Screening of DNA damage response and cell-cycle inhibitors identified several radiosensitizing targets, including DNA-PKcs, ATR, ATM, PARP, and HSP90, with DNA-PKcs inhibition showing particularly consistent effects across both radiation modalities [208]. These findings support the relevance of EBR-derived radiosensitization strategies for PRRT, while the observed differences between cell models emphasize the importance of tumor-specific responses.

#### 5.3.1. PARP Inhibitors

Poly (ADP-ribose) polymerase (PARP) is a family of proteins involved in several cellular processes, including DNA damage repair, chromatin remodeling, and programmed cell death. PARP-1 is one of the earliest proteins recruited to sites of DNA damage, where it binds to DNA breaks and facilitates repair by recruiting downstream repair proteins [209,210]. PARP is primarily involved in the repair of single-strand breaks through base excision repair, but also contributes to maintenance of genomic stability during repair of double-strand breaks (DSBs) through interactions with homologous recombination (HR) and non-homologous end joining (NHEJ) pathways. Inhibition of PARP results in accumulation of unrepaired DNA damage and enhanced sensitivity to ionizing radiation, making PARP inhibitors (PARPis) attractive radiosensitizing agents [211,212]. By preventing repair of radiation-induced DNA lesions, PARPis prolong and amplify DNA damage, thereby increasing the probability of tumor cell death. The magnitude of this effect is likely influenced by factors such as proliferation rate, DNA repair capacity, and cell cycle distribution.

The potential of PARP inhibition to enhance PRRT has been demonstrated in several preclinical studies (Table 4 and Table 5). An in vitro investigation on SSTR2-expressing rat pancreatic adenocarcinoma CA20948 cells and human osteosarcoma U2OS cells showed a potentiating effect of [^177^Lu]Lu-DOTATATE therapy when combined with the PARPi olaparib [132]. Combined treatment resulted in complete growth arrest compared with reduced proliferation after [^177^Lu]Lu-DOTATATE monotherapy. DNA DSBs persisted for up to six days after combination treatment, whereas DNA damage was largely repaired within four days after treatment with [^177^Lu]Lu-DOTATATE alone. The number of micronuclei, indicative of genomic instability and apoptosis, was also increased after combination treatment [132]. Similarly, Purohit et al. demonstrated that the cytotoxic effect of [^177^Lu]Lu-DOTATATE in BON and bronchopulmonary H727 cells was enhanced by the PARP inhibitors 1,5-dihydroxyisoquinoline (DHQ), PJ-34 and ABT-888 (veliparib). Co-treatment increased apoptosis, induced cell cycle arrest, and shifted cells into the sub-G1 phase, indicating enhanced cell death and impaired proliferation [133].

Additional support for PARP inhibition as a PRRT radiosensitization strategy was provided by Cullinane et al. using the PARPi talazoparib. In vitro, talazoparib significantly increased the number of DNA DSBs after [^177^Lu]Lu-DOTATATE treatment. In vivo, combination therapy in AR42J xenograft-bearing mice resulted in prolonged tumor growth inhibition, increased tumor necrosis, reduced tumor cell proliferation, and improved survival compared with [^177^Lu]Lu-DOTATATE alone (Table 4) [119]. Rauch et al. extended this concept to SSTR2-expressing small cell lung cancer models, combining [^177^Lu]Lu-DOTATOC with the PARPis olaparib or rucaparib [134]. Despite relatively low SSTR2 expression, PARP inhibition markedly increased PRRT potency in vitro, increased persistent DNA damage, delayed tumor growth, and improved survival in vivo, supporting the broader relevance of PARP inhibition as a radiosensitization strategy in SSTR-expressing neuroendocrine tumors.

Early clinical evidence supporting this strategy has emerged (Table 2). In the LuPARP phase I trial, Hallqvist et al. demonstrated that treatment with [^177^Lu]Lu-DOTATATE in combination with olaparib was feasible in patients with SSTR-positive tumors [104]. The most common adverse events were hematological toxicities, including lymphocytopenia, while non-hematological toxicities were generally mild and manageable. These results support clinical development of PARPi-PRRT combinations, although efficacy data remain limited. Since PARP inhibition may also increase radiation sensitivity in normal tissues, particularly bone marrow, hematological toxicity and treatment scheduling remain important considerations for further clinical development.

The encouraging safety results from LuPARP have led to several ongoing clinical studies evaluating PARP inhibition in combination with PRRT (Table 3). Multiple phase I and I/II trials are currently investigating [^177^Lu]Lu-DOTATATE together with olaparib in patients with GEP-NETs and other SSTR-positive malignancies (NCT04086485, NCT04375267, NCT05870423). In parallel, the PARP inhibitor talazoparib is being evaluated in combination with [^177^Lu]Lu-DOTATATE in patients with advanced grade 2 GEP-NETs (NCT05053854). These studies are expected to provide important information regarding optimal scheduling, safety, and preliminary efficacy of PARP inhibition in combination with PRRT. A pediatric phase II study is evaluating [^177^Lu]Lu-DOTATATE in combination with olaparib in children and adolescents with recurrent or relapsed SSTR-expressing solid tumors (NCT06607692), highlighting the growing interest in extending radiosensitization strategies beyond adult NET populations.

Altogether, PARP inhibition represents one of the most well-characterized strategies for enhancing PRRT through inhibition of DNA damage repair (Table 2, Table 3, Table 4, Table 5 and Table 7). Preclinical studies consistently demonstrate increased persistence of DNA damage and tumor control, and early clinical data indicate that the combination is feasible. Several ongoing phase I, I/II, and phase II trials are evaluating combinations of [^177^Lu]Lu-DOTATATE with olaparib or talazoparib across both adult and pediatric patient populations (Table 3), making PARP inhibition one of the most clinically advanced combination strategies under investigation. However, whether these combinations improve clinical outcomes compared with PRRT alone remains to be established.

#### 5.3.2. DNA-PK Inhibitors

DNA-dependent protein kinase (DNA-PK) is a serine/threonine protein kinase that plays a central role in the repair of DNA DSBs through the NHEJ pathway. Following radiation-induced DNA damage, DNA-PK is rapidly recruited to DSBs through interaction with the Ku70/Ku80 heterodimer, facilitating DNA end processing and ligation [213]. Because DSBs are particularly important for radiation-induced cell death, inhibition of DNA-PK has emerged as an attractive radiosensitization strategy [214].

The potential of DNA-PK inhibition as a strategy to enhance PRRT has been demonstrated by Waldeck et al., who performed a genome-wide CRISPR/Cas9 screen to identify determinants of sensitivity and resistance to [^177^Lu]Lu-DOTATATE therapy [120]. Among several DNA damage response pathways identified, genes involved in NHEJ were the most significantly enriched, with PRKDC, encoding DNA-PK, emerging as the strongest sensitization target. Genetic disruption of DNA-PK markedly increased sensitivity to [^177^Lu]Lu-DOTATATE, while pharmacological inhibition using the DNA-PK inhibitors nedisertib (peposertib) and AZD7648 enhanced the cytotoxic effects of PRRT in both SSTR2-overexpressing H1299 and AR42J cells. In vivo, combination treatment resulted in improved tumor control and prolonged survival compared with [^177^Lu]Lu-DOTATATE alone in SSTR-expressing xenograft models. The combination was effective even in models exhibiting relative resistance to PRRT, suggesting a potential role in overcoming treatment resistance.

Similar findings were reported by Reuvers et al., who demonstrated that DNA-PKcs inhibition sensitized NET cells to PRRT both in vitro and in vivo [121]. Treatment with DNA-PK inhibitors enhanced radiation-induced DNA damage and reduced tumor cell survival, supporting the concept that NHEJ represents a critical repair pathway following exposure to β-emitting radionuclides. No major acute hematological or renal toxicity was observed in the evaluated models, suggesting that a therapeutic window may exist for this strategy.

Mechanistically, inhibition of DNA-PK increases the persistence of radiation-induced DSBs, thereby promoting genomic instability, mitotic catastrophe, and apoptosis [214]. Since β-particle irradiation from [^177^Lu]Lu-DOTATATE continuously generates DNA damage over an extended period, suppression of DSB repair may be particularly effective for enhancing PRRT responses [215]. The identification of DNA-PK through an unbiased genome-wide screen suggests that NHEJ is an important determinant of intrinsic PRRT sensitivity and resistance [120].

Clinical translation of this strategy has already begun. The phase Ib clinical trial NCT04750954 is currently evaluating the DNA-PK inhibitor peposertib (nedisertib) in combination with [^177^Lu]Lu-DOTATATE in patients with SSTR-positive pancreatic NETs (Table 3). Results from this study will provide important information regarding safety, tolerability, and preliminary efficacy of DNA-PK inhibition in combination with PRRT.

DNA-PK inhibition is supported by direct mechanistic and therapeutic evidence in several preclinical PRRT models, including improved tumor control and survival. Clinical evidence is not yet available, but an ongoing phase Ib trial is evaluating the combination in patients with pancreatic NETs. Thus, DNA-PK inhibition represents a clinically emerging strategy for preventing repair of PRRT-induced DNA damage, although its safety and therapeutic benefit in patients remain to be established (Table 3, Table 4, Table 5 and Table 7).

### 5.4. Suppressing Cellular Stress Adaptation and Survival

In addition to activation of DNA repair pathways, tumor cells can respond to radiation-induced damage by activating cellular stress responses and survival signaling. These mechanisms may allow cells to adapt to oxidative stress, mitochondrial dysfunction, protein damage, and other consequences of irradiation, thereby reducing the therapeutic effect [204,205,216,217]. Inhibition of stress adaptation or pro-survival signaling therefore represents another strategy to increase tumor cell sensitivity to [^177^Lu]Lu-DOTATATE [3,7]. Several pathways involved in proteostasis, cellular metabolism, and growth and survival signaling have been investigated in combination with PRRT, although the strength of experimental and clinical evidence varies considerably between strategies.

#### 5.4.1. HSP90 Inhibitors

Heat shock protein 90 (HSP90) is a molecular chaperone involved in protein folding and stabilization, maintaining the function of numerous signaling proteins. In cancer, HSP90 supports the stability of oncogenic proteins and enables tumor cells to tolerate cellular stress and maintain signaling pathways that promote survival and proliferation [218]. HSP90 is overexpressed in several tumor types, including NETs, although no clear association with overall survival has been demonstrated [122].

Several HSP90 inhibitors (HSP90is) have been developed and evaluated in different tumor types, with variable outcomes [219]. In NET models, Hofving et al. demonstrated high HSP90 expression in the GOT1 siNET cell line and showed synergistic effects when combining HSP90 inhibitors with EBR in GOT1 cells, although similar effects were not observed in the P-STS cell line [122]. Corresponding experiments in primary siNET cells from patients confirmed enhanced responses when ganetespib was combined with EBR [122].

The radiosensitizing effects of HSP90 inhibition have also been demonstrated in the context of PRRT. In SSTR-expressing NET models, HSP90 inhibitors such as ganetespib and onalespib enhance the tumor-killing effect of [^177^Lu]Lu-DOTATATE, resulting in increased apoptosis and reduced tumor growth in both in vitro and in vivo models [122,123,135,220]. In vivo, combination treatment with [^177^Lu]Lu-DOTATATE and ganetespib resulted in greater tumor volume reduction in GOT1 xenografts compared with monotherapy [122]. Similarly, onalespib increased tumor doubling time when combined with [^177^Lu]Lu-DOTATATE in BON xenograft models, without altering radiopharmaceutical uptake or spatial distribution, indicating that the effect is mediated through radiosensitization rather than changes in tumor targeting [123].

Recent mechanistic work has clarified how HSP90 inhibition enhances PRRT efficacy. Engbers et al. demonstrated that ganetespib significantly increases radiation-induced cytotoxicity in NET cell models (GOT1 and BON1-SSTR2) when combined with both EBR and PRRT [136]. Notably, this radiosensitizing effect was not primarily driven by increased DNA damage or persistent double-strand breaks. Although homologous recombination was partially impaired, the overall contribution of DNA repair inhibition appeared modest. Instead, transcriptomic analyses revealed effects on stress-related pathways, including protein folding and heat shock responses, consistent with a broader disruption of proteostasis. These findings suggest that the radiosensitizing effect of HSP90 inhibition is not mediated by targeting a single pathway, but rather by a pleiotropic effect on multiple cellular stress processes.

On the whole, HSP90 inhibition has consistently enhanced the efficacy of [^177^Lu]Lu-DOTATATE in preclinical models, with recent mechanistic evidence indicating that the effect is primarily associated with disruption of protein homeostasis and cellular stress responses rather than direct inhibition of DNA repair. However, no clinical studies evaluating HSP90 inhibitors in combination with PRRT have yet been reported, and the effects of the combination on dose-limiting normal tissues remain insufficiently characterized (Table 3, Table 4, Table 5 and Table 7).

#### 5.4.2. mTOR Inhibitors

The mammalian target of rapamycin (mTOR) is a central regulator of cellular growth, metabolism, proliferation, and survival. mTOR signaling is activated downstream of several growth factor receptors, primarily through the phosphatidylinositol 3-kinase (PI3K)/AKT pathway [207,221,222,223]. Under physiological conditions, this pathway is negatively regulated by phosphatase and tensin homolog (PTEN), whereas dysregulation of PI3K/AKT/mTOR signaling contributes to tumor development and progression in several malignancies, including NETs [224]. Everolimus and temsirolimus are clinically available mTOR inhibitors (mTORis) that exert anti-proliferative and anti-angiogenic effects. Everolimus is approved for treatment of advanced NETs and has become an established component of systemic therapy in selected patients. In addition to direct effects on tumor cell proliferation, mTOR inhibition may influence tumor vasculature, cellular metabolism, and responses to radiation-induced stress, making mTORis potential candidates for combination with PRRT [7].

Preclinical studies have demonstrated anti-tumor effects of mTOR inhibition in NET models. In BON cells, treatment with the mTOR inhibitor RAD001 (everolimus) reduced proliferation and inhibited tumor cell growth [137,225]. In other tumor models, mTOR inhibition has also been shown to enhance responses to external beam radiation. For example, combining everolimus with EBR significantly inhibited tumor growth in a bladder cancer xenograft model compared with either treatment alone [226]. However, studies investigating mTOR inhibition in combination with PRRT have yielded mixed results. In the CA20948 rat pancreatic NET model, Pool et al. and Bison et al. evaluated combinations of everolimus and [^177^Lu]Lu-DOTATATE and found no clear improvement compared with PRRT alone. Furthermore, metastatic progression was observed in animals that failed to achieve complete remission, suggesting complex interactions between mTOR inhibition and tumor biology in this model [124,125]. Zellmer et al. evaluated the combination of everolimus and [^177^Lu]Lu-DOTATATE in AR42J pancreatic NET xenografts [126]. While PRRT significantly inhibited tumor growth, the addition of everolimus did not enhance the therapeutic response. The combination was generally well tolerated, with no major increase in hematological toxicity. Renal damage was observed but was considered primarily associated with PRRT rather than mTOR inhibition.

mTOR inhibition has also entered clinical evaluation in combination with PRRT (Table 2 and Table 3). In the phase I NETTLE study, Claringbold and Turner combined [^177^Lu]Lu-DOTATATE with everolimus in patients with advanced NETs [105]. The combination demonstrated encouraging anti-tumor activity, although hematological toxicity and nephrotoxicity required careful monitoring. Aljubran et al. reported additional clinical experience with everolimus and [^177^Lu]Lu-DOTATATE, demonstrating that the combination was feasible but associated with a relatively high frequency of grade ≥3 adverse events, including infection and neutropenia [106]. Ongoing clinical studies are investigating whether concomitant mTOR inhibition with everolimus can enhance the efficacy of [^177^Lu]Lu-DOTATATE in NET patients (NCT04665739 and NCT05773274) (Table 3).

Altogether, mTOR inhibition has a biological rationale as a strategy to suppress tumor cell proliferation and pro-survival responses during PRRT, and clinical feasibility of combining everolimus with [^177^Lu]Lu-DOTATATE has been demonstrated. However, direct preclinical studies not consistently shown improved therapeutic efficacy compared with PRRT alone, and clinical combination studies have reported hematological and renal toxicity. Thus, despite relatively advanced clinical evaluation, the therapeutic benefit of adding mTOR inhibition to PRRT remains uncertain, and ongoing trials will therefore determine whether selected patient populations may benefit from this combination (Table 2, Table 3, Table 4 and Table 7). Further, since both PRRT and mTOR inhibition may contribute to hematological toxicity, careful optimization of treatment schedules and dosing will likely be required.

#### 5.4.3. Multi-Target Receptor Tyrosine Kinase Inhibitors

Receptor tyrosine kinases (RTKs) regulate cellular proliferation, survival, angiogenesis, invasion, and metastasis. Dysregulation of RTK signaling contributes to tumor progression in many cancers, including NETs. Most receptor tyrosine kinase inhibitors (TKIs) investigated in NETs target vascular endothelial growth factor receptor (VEGFR) signaling either alone or together with kinases such as RET, MET, KIT, PDGFR, FGFR, and CSF1R. Their anti-tumor effects are therefore likely mediated through a combination of antiangiogenic, antiproliferative, and microenvironment-modulating mechanisms [7,227,228,229]. This is particularly relevant for newer agents such as surufatinib, which targets VEGFR1-3, FGFR1, and CSF1R, thereby influencing both angiogenesis and immune-cell populations within the tumor microenvironment [230].

Several multi-target RTK inhibitors have demonstrated clinical efficacy as monotherapies in NETs. Sunitinib is approved for treatment of pancreatic NETs, while cabozantinib, vandetanib, lenvatinib, surufatinib, pazopanib, and axitinib have shown anti-tumor activity in different NET subtypes [227,231,232]. The phase III CABINET trial demonstrated improved progression-free survival with cabozantinib in patients with advanced NETs, supporting RTK inhibition as a clinically relevant therapeutic strategy. Likewise, surufatinib and lenvatinib have shown promising activity in advanced NETs in phase II and III studies [230,233,234,235]. These findings establish RTK signaling as a clinically relevant therapeutic target in NETs, although they do not demonstrate benefit from combining TKIs with PRRT.

The strongest evidence supporting a radiosensitizing role of RTK inhibition in NETs comes from studies combining TKIs with EBR. In a patient-derived medullary thyroid carcinoma xenograft model (GOT2), combination treatment with vandetanib and EBR resulted in greater tumor volume reduction and prolonged time to tumor regrowth compared with either treatment alone. Similar results were observed for cabozantinib combined with EBR, leading to sustained tumor control throughout the follow-up period and a reduced proportion of animals reaching tumor progression [127] (Table 5). These findings suggest that inhibition of RTK signaling can enhance radiation responses in NET-related tumor models. In addition to receptor tyrosine kinase inhibition, downstream MAPK pathway inhibition may also be relevant in NETs. MEK inhibitors demonstrated preferential activity in pancreatic NET models in large-scale inhibitor screens, although their potential in combination with PRRT has not yet been established [122].

Potential mechanisms of radiosensitization include inhibition of pro-survival signaling pathways activated after radiation exposure, suppression or modulation of angiogenesis, alterations in tumor perfusion, modulation of DNA damage responses, and effects on the tumor immune microenvironment. However, despite strong biological rationale, direct evidence supporting combinations of TKIs with [^177^Lu]Lu-DOTATATE remains limited.

To date, no published preclinical studies have systematically evaluated [^177^Lu]Lu-DOTATATE in combination with clinically relevant TKIs such as sunitinib, lenvatinib, surufatinib, pazopanib, or axitinib. Likewise, no completed clinical studies have reported efficacy data for PRRT-TKI combinations (Table 2 and Table 4). Nevertheless, ongoing clinical trials are currently evaluating [^177^Lu]Lu-DOTATATE in combination with cabozantinib (NCT05249114) and sunitinib (NCT05687123) in patients with SSTR-positive NETs (Table 3). Since multi-target TKIs can also affect normal vasculature and glomerular function and are associated with treatment-related toxicities independent of PRRT, the tolerability of combined treatment will need to be evaluated in the ongoing clinical studies [236,237].

Multi-target RTK inhibitors have a biological rationale for combination with [^177^Lu]Lu-DOTATATE through effects on tumor growth and survival signaling, angiogenesis, and the tumor microenvironment. However, evidence for radiosensitization is currently derived mainly from EBR-based studies, and direct PRRT-specific evidence remains limited. Ongoing clinical trials will provide initial information on the safety and potential efficacy of combining TKIs with [^177^Lu]Lu-DOTATATE (Table 3, Table 4 and Table 7).

#### 5.4.4. Hedgehog Inhibitors

The Hedgehog (Hh) signaling pathway is an evolutionarily conserved developmental pathway involved in cellular differentiation, proliferation, stem-cell maintenance, and tissue regeneration. Aberrant activation of Hh signaling has been implicated in several malignancies, where it contributes to tumor growth, treatment resistance, and disease progression. In NETs, activation of Hh signaling has been associated with tumor regrowth, increased proliferative capacity, and radioresistance [238]. Consequently, pharmacological inhibition of this pathway has emerged as a potential strategy to enhance the efficacy of radiation-based therapies.

Several Hh inhibitors (Hhis) have been developed, including vismodegib (GDC-0449) and sonidegib (LDE225), both of which target the Smoothened (SMO) receptor, a key regulator of Hh signaling. In human medullary thyroid carcinoma tissue and MTC cell models, inhibition of Hh signaling using vismodegib demonstrated anti-tumor effects, supporting a functional role of the pathway in neuroendocrine malignancies [239]. Additional support for Hh inhibition in NETs was obtained in transgenic Rip1Tag2 mice bearing pancreatic islet-cell neoplasms. Treatment with sonidegib resulted in marked tumor growth inhibition, reducing tumor volume by approximately 95% and significantly prolonging survival compared with untreated controls [240]. These findings suggest that Hh signaling contributes to NET progression and may represent a therapeutically relevant target.

The only published study evaluating Hh inhibition in combination with PRRT in NETs was performed in the human small-intestinal NET xenograft model GOT1 [128] (Table 4). In this study, sonidegib monotherapy reduced tumor growth, while combination treatment with sonidegib and [^177^Lu]Lu-DOTATATE resulted in further reduction in tumor burden and prolonged time to tumor regrowth compared with either treatment alone. Gene expression analyses demonstrated significant modulation of several signaling pathways, including NF-κB signaling. Furthermore, expression of PDGFRA was increased after sonidegib treatment, whereas expression of PDGFA was reduced after [^177^Lu]Lu-DOTATATE and combination treatment [128]. These findings demonstrate that the enhanced therapeutic response was accompanied by changes in growth factor and cellular signaling pathways, although the mechanisms responsible for the combination effect remain incompletely understood.

Potential radiosensitization mechanisms of Hh inhibition include suppression of tumor cell survival signaling, inhibition of cancer stem-cell populations, reduction in tumor repopulation after irradiation, and modulation of the tumor microenvironment [241,242]. Hh inhibition has also been explored clinically in neuroendocrine-related malignancies such as small cell lung cancer, where sonidegib has been combined with standard cytotoxic therapy [243], although corresponding PRRT studies remain lacking.

In summary, Hh inhibition remains an underexplored strategy for enhancing [^177^Lu]Lu-DOTATATE therapy. Direct PRRT-specific evidence is currently limited to one preclinical GOT1 study demonstrating improved tumor control with combined sonidegib and [^177^Lu]Lu-DOTATATE treatment. No clinical studies evaluating Hh inhibitors in combination with PRRT have yet been reported. Additional studies are therefore needed to characterize the underlying mechanisms, effects on normal tissues, and potential clinical benefit of this strategy (Table 3, Table 4, Table 5 and Table 7).

#### 5.4.5. Nicotinamide Phosphoribosyltransferase (NAMPT) Inhibitors

NAMPT is the rate-limiting enzyme in the NAD+ salvage pathway and is important for maintaining cellular energy metabolism, redox balance, and DNA repair capacity [244]. Since tumor cells often have high metabolic demands and depend on NAD+ availability to tolerate cellular stress, NAMPT inhibition has been explored as a strategy to increase sensitivity to DNA-damaging therapies, including radiation.

The only published study evaluating NAMPT inhibition in combination with [^177^Lu]Lu-DOTATATE in NETs was performed in the human small-intestinal NET GOT1 model [129]. In vitro, the NAMPT inhibitor GMX1778 showed strong anti-tumor effects in GOT1 cells. In vivo, GMX1778 monotherapy inhibited tumor growth, while combined treatment with GMX1778 and [^177^Lu]Lu-DOTATATE resulted in a an improved anti-tumor response and markedly prolonged progression-free survival compared with either treatment alone [129].

Mechanistically, the effect of NAMPT inhibition is likely related to depletion of intracellular NAD+, resulting in impaired energy metabolism, reduced capacity to respond to radiation-induced stress, and compromised DNA repair [244,245]. However, NAMPT also has important functions in normal tissues, and systemic NAD+ depletion may narrow the therapeutic window. Therefore, toxicity and scheduling will be important considerations if this strategy is developed further.

At present, NAMPT inhibition remains an underexplored strategy for enhancing [^177^Lu]Lu-DOTATATE therapy. Direct PRRT-specific evidence is currently limited to one preclinical GOT1 study, in which combination treatment improved tumor control and progression-free survival. No clinical studies or ongoing clinical trials evaluating NAMPT inhibitors in combination with PRRT have yet been reported. Additional mechanistic and toxicity studies are required before the translational potential of this strategy can be assessed.

### 5.5. Promoting Tumor Cell Elimination

Following radiation-induced damage, the therapeutic outcome depends not only on the extent of the initial damage but also on whether tumor cells undergo irreversible growth arrest or cell death. Radiation can induce several forms of tumor cell elimination, including apoptosis, mitotic catastrophe, and immune-mediated cell killing, while surviving cells may recover or contribute to tumor regrowth [3,216,222,223]. Strategies that promote cell death or enhance immune-mediated elimination of irradiated tumor cells may therefore increase the therapeutic effect of [^177^Lu]Lu-DOTATATE. Such approaches include modulation of apoptosis and cell-cycle regulation, cytotoxic chemotherapy, and activation of anti-tumor immune responses, although these mechanisms often overlap with effects on DNA repair, receptor expression, and cellular stress responses.

Gene-expression studies in the GOT1 model have also demonstrated that [^177^Lu]Lu-DOTATATE and priming/fractionation affect pathways related to cell death and cell-cycle regulation [196,246,247]. These findings support the concept that the biological response to PRRT is dynamic and may provide opportunities to enhance tumor cell elimination through appropriately timed combination treatment.

#### 5.5.1. Immunotherapy

Immune checkpoint inhibitors (ICIs) have transformed the treatment of several malignancies by restoring anti-tumor immune responses through blockade of inhibitory pathways such as programmed cell death protein 1 (PD-1), programmed death ligand 1 (PD-L1), and cytotoxic T-lymphocyte-associated protein 4 (CTLA-4) [248]. However, the efficacy of ICIs in NETs has generally been modest, likely due to low tumor mutational burden, limited baseline immune infiltration, and a relatively immunosuppressive tumor microenvironment [249,250].

Radiation has long been recognized to modulate anti-tumor immunity, and combinations of EBR with ICIs have demonstrated synergistic effects in several tumor types [251,252]. More recently, attention has turned to targeted radionuclide therapies such as [^177^Lu]Lu-DOTATATE, which may similarly promote anti-tumor immune responses while simultaneously delivering tumor-selective radiation [253,254,255]. Potential mechanisms include increased tumor antigen release, enhanced antigen presentation, activation of inflammatory signaling pathways, modulation of immune-cell infiltration, and induction of immunogenic cell death. Together, these effects may increase susceptibility to immune checkpoint blockade and thereby improve therapeutic efficacy.

Preclinical evidence supporting this concept remains limited. The only published study directly evaluating PRRT-ICI combinations in NETs was performed by Esfahani et al. using the human pancreatic NET cell line QGP-1 implanted in immunodeficient mice reconstituted with human peripheral blood mononuclear cells [256]. Combination treatment with [^177^Lu]Lu-DOTATATE followed by pembrolizumab resulted in increased recruitment of cytotoxic immune cells, enhanced anti-tumor responses, and improved therapeutic efficacy compared with either monotherapy alone. These findings provide proof-of-principle that PRRT may sensitize NETs to immune checkpoint inhibition, although more representative immunocompetent NET models are still needed (Table 4).

Clinical experience with PRRT-ICI combinations remains limited but is growing (Table 2). Kim et al. reported the first phase I study combining [^177^Lu]Lu-DOTATATE with nivolumab in patients with lung neuroendocrine tumors, demonstrating that the combination was feasible with manageable toxicity, predominantly lymphopenia and other hematological adverse events [107]. Additional clinical experience has been reported in Merkel cell carcinoma, where combinations of PRRT and PD-1 blockade have induced durable responses in heavily pretreated patients [108,109]. Although Merkel cell carcinoma differs biologically from NETs, these studies support the feasibility of combining radionuclide therapy with immune checkpoint inhibition in SSTR-expressing malignancies.

Interest in this therapeutic strategy is reflected by several ongoing clinical trials (Table 3). Current studies are evaluating combinations of [^177^Lu]Lu-DOTATATE with nivolumab (NCT04525638), pembrolizumab (NCT03457948 and NCT05583708), avelumab (NCT04261855), and atezolizumab-containing regimens (NCT05142696) in patients with NETs, Merkel cell carcinoma, and small cell lung cancer. These trials will provide important information regarding safety, optimal treatment scheduling, and potential efficacy.

A key unanswered question is whether PRRT can increase immune activation in otherwise poorly immunogenic NETs and thereby increase their susceptibility to immune checkpoint blockade. In addition, the optimal sequencing of PRRT and immunotherapy remains unknown. Since PRRT may induce both immune stimulation and lymphocyte depletion, treatment timing may be critical for maximizing therapeutic benefit while minimizing immunosuppressive effects.

Collectively, combining PRRT with immune checkpoint inhibition has a biological rationale based on radiation-induced modulation of anti-tumor immunity. Direct preclinical evidence in NETs remains limited, while early clinical studies demonstrate feasibility and acceptable toxicity but do not yet establish improved efficacy compared with PRRT alone. Several ongoing clinical trials are evaluating different immune checkpoint inhibitors in combination with [^177^Lu]Lu-DOTATATE, and their results will help determine the clinical benefit, optimal sequencing, and safety of this strategy (Table 2, Table 3, Table 4 and Table 7).

#### 5.5.2. Chemotherapeutic Agents

Conventional chemotherapeutic agents are among the most extensively investigated combination partners for PRRT. Several cytotoxic drugs can enhance radiation-induced cell death through inhibition of DNA synthesis, interference with DNA repair, modulation of cell-cycle progression, depletion of nucleotide pools, or direct radiosensitization [195]. Because many of these agents are already used in the clinical management of NETs, combinations with [^177^Lu]Lu-DOTATATE represent one of the most clinically advanced strategies for enhancing PRRT efficacy [3] (Table 2, Table 3, Table 4, Table 5 and Table 7).

As discussed in Section 5.1.5, some chemotherapeutic agents, including temozolomide and gemcitabine, may also modulate SSTR expression and radiopharmaceutical uptake [190,191]. In addition to these effects on tumor targeting, chemotherapy may enhance the biological response to irradiation. In the patient-derived medullary thyroid carcinoma xenograft model GOT2, combination treatment with gemcitabine and [^177^Lu]Lu-DOTATATE prolonged time to progression compared with either treatment alone, although tumor growth inhibition was similar to that observed with gemcitabine monotherapy. Gemcitabine also enhanced responses to EBR in the same model, supporting its role as a radiosensitizer [130].

More recently, Cheng et al. investigated whether metronomic chemotherapeutics could enhance the efficacy of [^177^Lu]Lu-DOTATATE in vitro. Pretreatment with the ribonucleotide reductase inhibitors hydroxyurea, gemcitabine, and triapine increased SSTR2A expression, enhanced [^177^Lu]Lu-DOTATATE uptake, induced accumulation of cells in S-phase, and significantly reduced metabolic viability after PRRT compared with [^177^Lu]Lu-DOTATATE monotherapy. These findings suggest that modulation of nucleotide synthesis and cell-cycle progression may represent a strategy for enhancing PRRT responses and provide additional rationale for ongoing clinical evaluation of triapine in combination with PRRT [138].

The strongest evidence supporting chemotherapy-PRRT combinations comes from clinical studies (Table 2). Capecitabine, a prodrug of 5-fluorouracil, has been the most extensively investigated radiosensitizing agent. Multiple phase I and II studies demonstrated that combinations of [^177^Lu]Lu-DOTATATE or [^177^Lu]Lu-DOTATOC with capecitabine are feasible and generally well tolerated, with encouraging response rates and prolonged progression-free survival [60,110,111,112,113,118]. Combination treatment with capecitabine and temozolomide (CAPTEM) has shown particularly promising activity. Objective response rates exceeding those typically reported for PRRT alone have been observed in several studies, particularly in pancreatic NETs and FDG-avid tumors [113,116,117].

Additional support for PRRT-based chemoradionuclide therapy was provided by Kong et al. and Kashyap et al., who combined [^177^Lu]Lu-DOTATATE with 5-fluorouracil and reported favorable long-term outcomes and manageable toxicity profiles [114,115]. Prospective studies evaluating CAPTEM combined with PRRT have reported encouraging efficacy [62,63]. More recently, the randomized phase II AGITG CONTROL NETs trial evaluated concurrent CAPTEM and [^177^Lu]Lu-DOTATATE in patients with advanced pancreatic and small bowel NETs [64]. While no progression-free survival benefit was observed in patients with small bowel NETs compared with PRRT alone, encouraging activity was observed in pancreatic NETs, providing the first randomized prospective evidence supporting further evaluation of PRRT-based chemoradionuclide therapy in this subgroup. However, not all studies have demonstrated a clear clinical benefit from chemotherapy-based radiosensitization. In the multicenter randomized phase II trial reported by Becx et al., the addition of capecitabine to [^177^Lu]Lu-DOTATATE did not significantly improve objective response rate, progression-free survival, or overall survival compared with PRRT alone, although the study was closed prematurely due to slow recruitment [65]. Collectively, these findings suggest that the benefit of chemotherapy-based radiosensitization may depend on both the chemotherapeutic regimen and the underlying NET subtype, emphasizing the need for adequately powered phase III studies.

Several ongoing clinical trials reflect the continued interest in chemotherapy-based combination strategies (Table 3). Current studies are evaluating combinations of [^177^Lu]Lu-DOTATATE or [^177^Lu]Lu-DOTATOC with capecitabine, temozolomide, or CAPTEM (NCT04194125, NCT05247905, NCT07185672, and NCT05387603). Inhibition of ribonucleotide reductase has emerged as a novel approach. Triapine impairs deoxyribonucleotide synthesis and may enhance radiation-induced DNA damage by limiting DNA repair capacity. The multicenter phase I ETCTN 10388 study evaluated triapine in combination with [^177^Lu]Lu-DOTATATE in patients with well-differentiated NETs and established 150 mg oral triapine combined with 7.4 GBq [^177^Lu]Lu-DOTATATE as the recommended phase II dose. Preliminary anti-tumor activity was observed, although grade ≥3 hematological adverse events were frequent [80]. Further clinical evaluation of this combination is ongoing (NCT05724108). Since both PRRT and cytotoxic chemotherapy may contribute to hematological toxicity, treatment schedule, cumulative treatment exposure, and long-term bone marrow toxicity are important considerations when combining these approaches [257,258,259,260].

Among the strategies reviewed, chemotherapeutic agents represent one of the most clinically mature combination approaches for PRRT, with substantial clinical experience using capecitabine, 5-fluorouracil, and CAPTEM. Preclinical studies demonstrate several potential mechanisms, including modulation of SSTR expression, inhibition of DNA synthesis and repair, cell-cycle redistribution, and increased tumor cell killing. However, randomized clinical studies have not consistently demonstrated improved outcomes compared with PRRT alone, and the benefit may differ according to chemotherapy regimen and NET subtype. Ongoing randomized trials will help define which patients, if any, benefit from PRRT-based chemoradionuclide therapy and how these combinations should be scheduled (Table 2, Table 3, Table 4, Table 5 and Table 7).

#### 5.5.3. p53 Stabilization

The tumor suppressor p53 plays a central role in the cellular response to DNA damage by regulating cell-cycle arrest, DNA damage responses, and apoptosis. Following radiation-induced DNA damage, stabilization and activation of p53 can promote elimination of damaged cells, whereas suppression of p53 signaling may contribute to treatment resistance. Pharmacological stabilization of p53 may therefore provide a strategy to enhance radiation-induced tumor cell death.

This approach has been investigated in combination with [^177^Lu]Lu-DOTATATE using the p53-stabilizing peptide VIP116, which inhibits the negative regulators MDM2 and MDM4. In neuroblastoma spheroid models, VIP116 potentiated the effects of [^177^Lu]Lu-DOTATATE, with synergistic growth inhibition observed in IMR-32 spheroids [139]. Combination treatment increased p53 levels and downstream expression of p21, Bax, and cleaved caspase-3, supporting increased p53-mediated cell-cycle regulation and apoptosis. The response varied between models and was associated with p53 status and differences in MYCN signaling. The combination was subsequently evaluated in vivo in mice bearing neuroblastoma xenografts. VIP116 and [^177^Lu]Lu-DOTATATE monotherapies both delayed tumor growth and prolonged survival, while combination treatment resulted in further improvement in progression-free survival and an increased proportion of complete remissions. No significant nephrotoxicity was observed [131]. Although these studies provide direct evidence that p53 stabilization can enhance [^177^Lu]Lu-DOTATATE-induced tumor cell elimination, the available evidence is restricted to preclinical neuroblastoma models and may depend on preservation of functional wild-type p53 signaling. Clinical studies evaluating p53 stabilization in combination with PRRT have not yet been reported.

#### 5.5.4. Epigenetic Modulation of Radiation Response

As discussed in Section 5.1.4, epigenetic-modifying drugs can increase SSTR expression and thereby influence radiopharmaceutical uptake. However, epigenetic regulation also affects several processes involved in the cellular response to radiation, including chromatin accessibility, DNA repair, apoptosis, and stress response signaling [261]. Epigenetic modifiers may therefore enhance PRRT through mechanisms that are partly independent of their effects on SSTR expression.

HDAC inhibition promotes a more open chromatin structure and altered gene expression, which may further influence cellular responses to radiation [262]. HDAC inhibition may additionally enhance radiation sensitivity through effects on DNA damage and repair. HDAC inhibitors have been shown to induce DNA damage and impair cellular DNA repair mechanisms, thereby increasing sensitivity to radiation-induced damage [263,264]. In GOT1 cells, VPA induced growth inhibition, activation of death-receptor signaling, and mitochondria-mediated apoptosis, accompanied by activation of caspase-3/7 and altered expression of several apoptosis-related genes [184]. VPA has also been shown to modulate TGF-β and NOTCH signaling in NET models, indicating broader effects on pathways involved in differentiation and proliferation [184,265]. In a larger inhibitor screen, small-intestinal NET models were more sensitive to HDAC inhibition than pancreatic NET models, suggesting that responses to epigenetic treatment may differ between NET subtypes [102].

DNMTis have demonstrated radiosensitizing effects in several experimental models. The cytidine analog zebularine, a DNMT inhibitor, enhanced sensitivity to both external irradiation and radiopharmaceutical therapy in vitro, although these studies were performed in non-NET models [266]. In BON and QGP-1 cells, the DNMTi decitabine combined with 5-fluorouracil increased apoptosis, reduced proliferation, and enhanced radiosensitivity [140]. Other epigenetic regulators may also influence radiation sensitivity. In a large-scale radiosensitizer screen, the BET inhibitor I-BET151 synergized with external radiation in GOT1 and P-STS cells [122]. However, this evidence is derived from EBR, and no studies evaluating BET inhibition in combination with PRRT have yet been reported.

Altogether, epigenetic modifiers may enhance PRRT through effects at several stages of the treatment response, including SSTR upregulation and modulation of cellular radiation responses. However, direct evidence for epigenetic radiosensitization of PRRT remains limited, and the effects on dose-limiting normal tissues are insufficiently characterized. Further studies are needed to determine whether epigenetic modulation can improve the therapeutic effect of PRRT independently of changes in tumor targeting.

## 6. Conclusions and Outlook

[^177^Lu]Lu-DOTATATE has become an established and effective treatment for patients with SSTR-positive NETs [11,12,13]. Nevertheless, complete and durable responses remain relatively uncommon, highlighting the need for strategies that improve therapeutic efficacy while preserving the favorable safety profile of PRRT [3,11,14,15]. As reviewed here, the therapeutic effect of PRRT depends on several consecutive and interconnected steps, from SSTR expression and radiopharmaceutical uptake to tumor absorbed dose, induction and repair of radiation damage, cellular stress responses, and ultimately tumor cell elimination (Figure 1). Treatment optimization may therefore target different stages of this process, including increasing receptor availability and tumor targeting, improving radiopharmaceutical delivery and dosimetry, preventing repair of radiation-induced damage, suppressing cellular stress adaptation and survival, and promoting tumor cell elimination [3,34].

Available studies demonstrate that several of these approaches can improve individual components of the treatment response, although effects at one stage do not necessarily translate into improved therapeutic efficacy. Strategies including radiation priming, epigenetic modulation, and selected pharmacological agents can increase SSTR expression or radiopharmaceutical uptake, but increased receptor expression does not consistently result in increased tumor uptake or improved therapeutic efficacy [34,179,180,185,186,187,190,196]. Similarly, strategies that inhibit DNA repair or cellular survival responses may increase tumor cell sensitivity to radiation but may also affect normal tissues [3,7]. Treatment-enhancement strategies should therefore be evaluated according to their effects on the complete therapeutic chain, including tumor targeting, absorbed dose, biological response, and toxicity.

The level of evidence varies considerably between strategies. PARP and DNA-PK inhibition have shown consistent preclinical radiosensitization and have entered early clinical evaluation [104,119,120,121,132]. HSP90 inhibition has likewise enhanced PRRT efficacy in several preclinical models [122,123,136], while chemotherapy-based approaches, particularly capecitabine, temozolomide, and CAPTEM, have generated the largest body of clinical evidence, although randomized studies have not consistently demonstrated improved outcomes compared with PRRT alone [62,63,65,111,112,113,114,115,116,117,118]. Other strategies, including mTOR inhibition, Hedgehog inhibition, NAMPT inhibition, immunotherapy, multi-target receptor tyrosine kinase inhibitors, p53 stabilization, and epigenetic modulation, are supported by different levels of preclinical or early clinical evidence and require further evaluation before their role in PRRT optimization can be established [105,106,126,127,128,129,256].

An important observation is that several of the most promising combination partners are already clinically available and routinely used in oncology, potentially facilitating rapid clinical translation [3,7]. Clinical trials evaluating combinations of PRRT with PARP inhibitors, DNA-PK inhibitors, immune checkpoint inhibitors, chemotherapeutic agents, mTOR inhibitors, and tyrosine kinase inhibitors are currently ongoing and will help define their future role in NET management (Table 3).

A central challenge for future combination strategies will be to identify which step of the therapeutic response limits treatment efficacy and to select treatment accordingly. Tumors with low or heterogeneous SSTR expression may primarily benefit from strategies that increase receptor availability or radiopharmaceutical uptake, whereas tumors with sufficient uptake but limited treatment response may require approaches targeting DNA repair, cellular stress adaptation, or tumor cell elimination [3,7,34]. The optimal strategy may also differ between tumor types and individual patients, reflecting differences in SSTR expression, tumor differentiation/grade and proliferation, molecular characteristics, prior treatments, and intrinsic radiation sensitivity.

Importantly, improved tumor response must be considered together with effects on normal tissues. Combination approaches targeting DNA repair, apoptosis regulation, or cellular stress responses may also increase hematological or renal toxicity, and the therapeutic window may be further influenced by patient-related factors such as age, comorbidities, prior treatments, and baseline organ function [3,88,89,90,91,92,94,95,96,97,99,100,267]. Careful optimization of treatment scheduling, dosing, and therapeutic combinations will therefore remain important as these strategies are translated into broader patient populations, including children and adolescents.

In addition to the strategies already evaluated in combination with PRRT, several other candidate radiosensitization targets deserve future investigation. DNA damage response pathways involving ATM, ATR, CHK1, and other cell-cycle checkpoint regulators are potential candidates because of their established roles in radiation sensitivity, DNA damage signaling, and DNA repair [215]. Nuclear export regulators such as XPO1/CRM1 may similarly enhance radiation responses through modulation of DNA damage response and stress response pathways [268,269]. Although direct evidence in NET models and PRRT remains limited, these approaches are currently being investigated in combination with external beam radiotherapy and other DNA-damaging therapies and may represent future PRRT combination partners.

Additional opportunities may arise from targeting mitotic checkpoint regulation. Inhibitors of Aurora kinases, PLK1, MPS1, CENP-E, and kinesin spindle protein (KSP) can disrupt chromosome segregation and mitotic progression, thereby promoting mitotic catastrophe following radiation-induced DNA damage [270]. These agents have demonstrated radiosensitizing properties in several tumor types and may therefore warrant evaluation in combination with PRRT [271].

Modulation of apoptotic signaling pathways also represents another potential strategy. Since many well-differentiated NETs retain wild-type TP53, resistance to therapy may in part reflect impaired execution of apoptosis rather than defective DNA damage sensing [272,273]. Agents that lower the apoptotic threshold, including BH3 mimetics targeting BCL-2 family proteins, SMAC mimetics, death receptor agonists, and inhibitors of c-FLIP-mediated survival signaling, may therefore increase susceptibility to PRRT-induced cell death by facilitating apoptosis in response to radiation-induced stress [274,275,276,277]. Supporting this concept, pharmacological stabilization of p53 enhanced the efficacy of [^177^Lu]Lu-DOTATATE and increased apoptosis in preclinical neuroblastoma models [131,139], providing initial support for targeting apoptosis regulation in combination with PRRT.

Epigenetic regulation may provide additional targets for future combination strategies. BET and EZH2 inhibitors, as well as other compounds identified in large-scale inhibitor screens of NET models, can modulate chromatin accessibility and transcriptional programs relevant to tumor cell survival and treatment response [122,278,279,280,281]. BET inhibition has demonstrated radiosensitizing effects with EBR in NET models, although direct evidence in combination with PRRT is lacking. Collectively, these approaches highlight the broad range of molecular pathways that remain to be explored for enhancement of [^177^Lu]Lu-DOTATATE therapy.

Taken together, the available evidence supports the concept that PRRT efficacy can be improved by targeting several interconnected steps of the therapeutic process, from receptor availability and radiopharmaceutical uptake to tumor absorbed dose and the subsequent biological response to radiation. Rather than being competing approaches, strategies acting at different stages of this process are likely complementary. Future treatment optimization may therefore involve combinations that simultaneously increase tumor targeting and absorbed dose, limit cellular repair or survival responses, and promote tumor cell elimination. Continued integration of molecular biology, radiobiology, dosimetry, and translational research will be essential for identifying combination strategies that improve the therapeutic window and determining how they can be implemented safely in patients. The growing number of ongoing clinical trials suggests that the next generation of PRRT may increasingly rely on rational combination therapies to improve outcomes for patients with NETs [3,7,34].

## Figures and Tables

**Table 1:** Clinical experience with [^177^Lu]Lu-DOTATATE and [^177^Lu]Lu-DOTATOC therapy in NETs. Data represent PRRT monotherapy; where studies evaluated combination treatment, only data from the PRRT monotherapy arm are presented.

Administered Activity (GBq/Cycle) (Number of Cycles)	No. of Patients	CR (%)	PR (%)	MR (%)	SD (%)	OS (Months)	PFS (Months)	Toxicity/Side Effects	Reference
3.7–7.4 (7 cycles) DOTATATE	35	3	35	0	41	NA	NA	0% anemia (grade 3), 1% leukocytopenia, 1% thrombocytopenia	Kwekkeboom et al., 2003 [42]
3.7–7.4 (4 cycles) DOTATATE	504 (toxicity analysis), 310 (efficacy analysis)	2	28	16	35	46	33	3.6% hematologic toxicity (≥grade 3), myelodysplastic syndrome (three patients), liver toxicity (two patients)	Kwekkeboom et al., 2008 [43]
8.0 (1–5 cycles) DOTATATE	26	0	38	NA	50	NA	NA	12% hematologic toxicity (grade 3)	Swärd et al., 2010 [54]
3.7–7.4 (6 cycles) DOTATATE	51	2	27	21	27	NA	36	No major acute or delayed renal or hematologic toxicity leukopenia (grade 3), thrombocytopenia	Bodei et al., 2011 [44]
8.0 (6–9 cycles) DOTATATE	68	0	60	28	13	53	34	5.9% hematologic toxicity (grade 3), 1.7% leucopenia, 2.2% thrombocytopenia, 0.9% anemia	Ezziddin et al., 2014 [47]
7.9 (4 cycles) DOTATATE	74	0	36	18	35	55	26	NA	Ezziddin et al., 2014 [46]
7.9 (4 cycles) DOTATATE	61	0	13	31	48	61	33	8.2% hematological toxicity (≥grade 3)	Sabet et al., 2015 [52]
3.7–5.5 (5 cycles) DOTATATE	43	7	NA	NA	77	NA	36	Nausea (max grade 2), asthenia, mild alopecia	Paganelli et al., 2014 [51]
7.0 (1–4 cycles) DOTATOC	56	16	18	NA	32	34	17	1.7% grade 3 myelotoxicity, 20% mild renal toxicity	Baum et al., 2016 [18]
3.7–5.5 (4–5) DOTATATE	34	3	12	NA	47	49	18	No acute or delayed hematologic toxicity	Ianniello et al. 2016 [50]
7.4 (4 cycles) DOTATATE	116	1	17	64		48	20	1% neutropenia (≥grade 3), 2% thrombocytopenia, No cases of MDS or acute leukemia	Strosberg et al., 2017 and 2021 [12,13]
5.6–7.4 (4 cycles) DOTATATE	143	0	8	NA	46	NA	NA	1% MDS and liver toxicities	Hamiditabar et al., 2017 [49]
3.7 (1–6 cycles) DOTATAT	443	2	37	NA	43	63	29	0.7% leukemia, 1.5% MDS	Brabander et al., 2017 [11]
7.4 (4 cycles) DOTATATE	79	NA	6	NA	61	48	48	Mild decreases in platelets, leukocytes, hemoglobin; no grade 3–4 events No significant nephrotoxicity Fatigue and diarrhea in a few patients	Ballal et al., 2017 [60]
3.7 (5 cycles)	28	11	24	NA	43	NR	53	In 3.7 group, 39.2% patients had anemia, 10.7% thrombocytopenia, 21.4% leucopenia.	Sansovini et al., 2017 [53]
5.5 (5 cycles) DOTATATE	32	3	16	NA	59	64	22	In 5.5 Group 31.3% patients had anemia, 28.1% thrombocytopenia, 21.9% leucopenia, no grade 3 or 4 hematological	
7.4 (4 cycles) DOTATATE	200	1	24	NA	68	43	27	1.5% acute leukemia, 0.5% chronic leukemia 15% grade 3 or 4 bone marrow toxicity. 4% grade 2 kidney toxicity 0.5% grade 4 kidney toxicity	Garske-Román et al., 2018 [48]
7.4 (4 cycles) DOTATATE	52	0	23	36	33	NR	16	10.0% subacute grade 3 or 4 toxicities, 52% lymphocytopenia, 33% nausea, 30.8% fatigue	Del Prete et al., 2019 [45]
7.4 (4 cycles) DOTATATE	34	3	56	NA	24	NA	18	12% Subacute hematological toxicity, grade 3 or 4.9% hormonal crisis	Zandee et al., 2019 [56]
7.4 (4 cycles) DOTATATE	30	NA	23	NA	67	NR	30	20% hematological toxicity, grade 3 or 4,7% catecholamine release	Zandee et al., 2019 [57]
7.4 (1–10 cycles) DOTATATE	102	4	45	NA	44	24	42	10.8% Bone marrow toxicity grade 3–4 occurred, 1.0% acute myeloid leukemia	Fröss-Baron et al., 2021 [55]
7.4 (1–4 cycles) DOTATATE	48	NA	44	NA	44	59	23	14% lymphopenia, no incidence of MDS or leukemia	Zidan et al., 2022 [58]
7.4 (4 cycles) DOTATATE	36	0	31	NA	NA	NR	31	Generally well tolerated. Grade ≥3 adverse events were mainly hematological. No grade ≥3 nephrotoxicity, myelodysplastic syndrome, or leukemia were observed.	Satapathy et al., 2025 [61]
7.4 (4 cycles) DOTATATE	162	NA	NA	NA	NA	NA	NA	NA	Puranik et al., 2025 [62]
7.4 (3–5 cycles) DOTATATE	10	7	0	NA	29	21	10	One patient showed transient grade 3 anemia, grade 4 thrombocytopenia, another patient showed only transient G4anemia and G3 thrombocytopenia	di Santo et al., 2026 [63]
7.4 (4 cycles) DOTATATE	13	0	23	NA	69	NR	51	Grade 3–4 adverse events were mainly lymphopenia. Long-term hematologic toxicity occurred in 31% of patients. One case of treatment-related acute leukemia was reported. No late renal toxicity was observed.	Chan et al., 2026 [64]
7.4 (4 cycles) DOTATATE	61	2	44	NA	49	61	32	Grade ≥3 adverse events occurred in 18% of patients, mainly hematological and gastrointestinal toxicities.	Becx et al., 2026 [65]
7.4 (4 cycles) DOTATATE	21	2	12	NA	6	NR	37	Generally well tolerated. Grade 3 hematological toxicity was uncommon (primarily lymphopenia and anemia). No severe renal toxicity or treatment-related deaths were observed. One case of CML occurred during long-term follow-up.	Kobayashi et al., 2026 [66]
7.4 (4 cycles) DOTATATE	10	0	30	NA	70	NR	NR	Generally well tolerated. Grade ≥3 treatment-related adverse events were primarily hematological, most commonly lymphopenia and thrombocytopenia. No cases of AML, MDS, severe infections, or other secondary malignancies were reported.	Ma et al., 2026 [67]
5.5–6.3 (4–6 cycles) DOTATATE	20	0	10	NA	50	NR	12	Generally well tolerated. No grade ≥3 hematological toxicity. Mild transient gastrointestinal symptoms and fatigue were common. One patient developed grade 1 nephrotoxicity. No hepatotoxicity was observed.	Agrawal et al., 2026 [68]
7.4 (4 cycles) DOTATATE	41	0	26	NA	34	56	21	Grade 3–4 AEs: 44%; most common were neutropenia (5%) and hypertension (10%); one treatment-related death (acute leukemia)	Baudin et al., 2026 [69]

Abbreviations: NA = not available, NR = not reached, CR = complete response, PR = partial response, MR = minor response, PFS = progression-free survival, SD = stable disease, OS = overall survival, MDS = myelodysplastic syndrome.

**Table 2:** Clinical studies evaluating combination strategies for enhancement of [^177^Lu]Lu-DOTATATE therapy in NETs.

Strategy Combination	Treatment Combination Administered Activity (GBq per Cycle) (Number of Cycles)	No. of Patients	Reported Tumor Response						Toxicity/Side Effects	Reference
			CR (%)	PR (%)	MR (%)	SD (%)	OS (Months)	PFS (Months)		
PARP inhibitors										
	7.4 (4 cycles) DOTATATE + Olaparib	18	3	NA	8	NA	NA	NA	Hematological toxicity (including grade 3–4 lymphocytopenia) was most common; non-hematological effects were mainly mild (nausea, fatigue), with occasional liver enzyme elevations.	Hallqvist et al., 2025 [104]
mTOR inhibitors										
	7.8 (1–4 cycles) DOTATATE + Everolimus	16	0	44	NA	56	NR	NR	grade 2 or 3 neutropenia and thrombocytopenia grade 2 nephrotoxicity	Claringbold et al., 2015 [105]
	3.7–7.4 (4 cycles) DOTATATE + Everolimus	11	NA	9	NA	82	NA	23	grade ≥3 events in ~36% of patients (including infection and neutropenia), occasional dose reductions, and one serious thrombotic event.	Aljubran et al., 2024 [106]
Checkpoint Inhibitors										
	3.7–7.4 (4 cycles) DOTATATE + Nivolumab	9	14	NA	NA	NA	NA	NA	lymphopenia (n = 7), thrombocytopenia (n = 4), anemia (n = 3), and nausea (n = 3). The most common grade 3 TRAE was lymphopenia (n = 4).	Kim et al., 2020 [107]
	3.5 (1–6 cycles) DOTATATE + PD-1	2	NA	NA	NA	NA	NA	NA	lymphocyte counts decreased	Aicher et al., 2022 [108]
	7.4 (4 cycles) DOTATATE + Ipilimumab and nivolumab	1	NA	NA	NA	NA	NA	NA	Transient hemtoxicity and mild nausea	Ferdinandus et al., 2022 [109]
Chemotherapy drugs										
	7.4 (4 cycles) DOTATATE + Capecitabine	7	0	14	NA	NA	NA	NA	1 × grade-3 anemia; one case of grade-3 thrombocytopenia; one occurrence of grade-2 thrombocytopenia; no grade-4 hematologic events were observed.	van Essen et al., 2008 [110]
	7.4 (4 cycles) DOTATATE + Capecitabine	27	NA	NA	NA	NA	36	NA	No hematologic toxicity, no MDS, leukemia, or late renal failure	Hubble et al., 2010 [111]
	7.8 (4 cycles) DOTATATE + Capecitabine	33	0	24	NA	70	NR	NR	1 × Grade-3 thrombocytopenia; no neutropenia; no nephrotoxicity	Claringbold et al., 2011 [112]
	7.8 (4 cycles) DOTATATE + Capecitabine and Temozolomide	35	13	70	NA	38	NR	48	18% of cases had moderate nausea, and 3% had severe nausea. 24% had moderate low platelet levels, and 6% had severe low white blood cell levels. There were no very severe side effects, and no kidney or liver problems were reported.	Claringbold et al., 2012 [113]
	7.4 (3–6 cycles) DOTATATE + 5-FU	63	0	30	9	29	>60	NA	NA	Kong et al., 2014 [114]
	6.0–10 (3–5 cycles) DOTATATE + 5-FU	52	2	28	NR	68	NR	48	well tolerated with negligible grade 3/4 toxicities	Kashyap et al., 2015 [115]
	7.9 (4 cycles) DOTATATE + Capecitabine and Temozolomide	30	13	70	NA	20	NR	48	transient nausea of grade 2, grade 3 Hematological toxicity thrombocytopenia (10%) and anemia (10%).	Claringbold et al., 2016 [116]
	5.5–7.4 (6–8 cycles) DOTATATE + Capecitabine	88	0	34	9.1	50	NR	NR	No clinical thrombocytopenia; no grade 3–4 events. Two patients had mild insufficiency; no significant nephrotoxicity. Diarrhea and anorexia occurred in a few patients.	Ballal et al., 2017 [60]
	7.1 (3 cycles) DOTATATE + Temozolomide ± Capecitabine	15 (3 + 12)	NA	NA	NA	NA	25	7	Grade 4 hepatic failure: 1 patient Grade 3 hepatic toxicity (liver enzymes): 4 patients (27%)	Yordanova et al., 2019 [117]
	5.5 (8 cycles) DOTATATE + Capecitabine	33	0	30	NA	55	NR	31	Grade 3 (G3) or 4 (G4) hematological toxicity occurred in 16.2% of patients. diarrhea in 5.4% of cases and asthenia in 5.4%. No renal toxicity was observed for the duration of follow-up	Nicolini et al., 2021 [118]
	7.4 (4 cycles) DOTATATE + Capecitabine	36	0	33	NA	NA	NR	29	Generally well tolerated. Grade ≥3 adverse events were mainly hematological. No grade ≥3 nephrotoxicity, myelodysplastic syndrome, or leukemia were observed.	Satapathy et al., 2025 [61]
	7.4 (4 cycles) DOTATATE + Temozolomide and Capecitabine	162	NA	NA	NA	NA	NA	NA	NA	Puranik et al., 2025 [62]
	7.4 (3–5 cycles) DOTATATE + Temozolomide ± Capecitabine	7 7	00	71 14	NA NA	029	NR 21	42 13	In the temozolomide group, some grade 3–4 hematological and renal toxicities were reported, whereas no severe (grade 3–4) hematological or renal toxicities were observed in the CAPTEM group.	di Santo et al., 2026 [63]
	7.4 (4 cycles) DOTATATE + Temozolomide and Capecitabine (pNET and siNET)	18 32	00	72 34	NA NA	28 63	NR NR	59 46	Grade ≥3 adverse events were primarily hematological and consistent with the known safety profile of CAPTEM and PRRT.	Chan et al., 2026 [64]
	7.4 (4 cycles) DOTATATE + Capecitabine	50	2	30	NA	60	76	46	Grade ≥3 adverse events occurred in 26% of patients, mainly hematological and gastrointestinal toxicities.	Becx et al., 2026 [65]
	7.4 (4 cycles) DOTATATE + Triapine	28	0	6	NA	19	NA	NR	Grade ≥3 treatment-related AEs in 77%; lymphopenia (58%), leukopenia (39%), neutropenia (35%), anemia (23%); most cytopenias were transient	Chauhan et al., 2026 [80]

Abbreviations: NA = not available, NR = not reached, CR = complete response, PR = partial response, MR = minor response, PFS = progression-free survival, SD = stable disease, OS = overall survival, MDS = myelodysplastic syndrome.

**Table 3:** Ongoing clinical trials evaluating [^177^Lu]Lu-DOTATATE or [^177^Lu]Lu-DOTATOC in combination with other agents in patients with SSTR-expressing tumors.

Class of Combined Agent	Trial Number	Treatment Combination	n	Patients	Study Phase	Status
PARP inhibitors	NCT04086485	[^177^Lu]Lu-DOTATATE + Olaparib	56	GEP-NET	1/2	Recruiting
	NCT05870423	[^177^Lu]Lu-DOTATATE + Olaparib	24	mNET	1	Recruiting
	NCT04375267	[^177^Lu]Lu-DOTATATE + Olaparib	18	SSRT+	1	Active, not recruiting
	NCT05053854	[^177^Lu]Lu-DOTATATE + Talazoparib	24	GEPNET, G 2	1	Recruiting
	NCT06607692	[^177^Lu]Lu-DOTATATE + Olaparib	25	Children and adolescents with recurrent or relapsed solid tumor expressing SSTR	2	Recruiting
DNA-PK inhibitors	NCT04750954	[^177^Lu]Lu-DOTATATE + Peposertib	29	pNET	1	Active, not recruiting
mTOR inhibitors	NCT03049189	[^177^Lu]Lu-DOTATOC + Everolimus	309	SSTR+/GEP-NET	3	Active. not recruiting
	NCT04665739	[^177^Lu]Lu-DOTATATE + Everolimus	70	bronchial NET	2	Recruiting
	NCT05773274	[^177^Lu]Lu-DOTATATE + Everolimus	100	NET	2	Recruiting
TK inhibitors	NCT05249114	[^177^Lu]Lu-DOTATATE + Cabozantinib	6	SSTR+ NET	1	Active, not recruiting
	NCT05687123	[^177^Lu]Lu-DOTATATE + Sunitinib malate	24	pNET	1	Recruiting
Immune checkpoint inhibitors	NCT04525638	[^177^Lu]Lu-DOTATATE + Nivolumab	30	NET G3 or NEC	2	Unknown status
	NCT04261855	[^177^Lu]Lu-DOTATATE + Avelumab	19	mMCC	1/2	Recruiting
	NCT03457948	[^177^Lu]Lu-DOTATATE + Pembrolizumab	32	NET G2 and G3	2	Active, not recruiting
	NCT05583708	[^177^Lu]Lu-DOTATATE + Pembrolizumab	18	Merkel Cell Cancer	2	Recruiting
	NCT05142696	[^177^Lu]Lu-DOTATATE + Carboplatin, Etoposide and Atezolizumab	24	Extensive Stage SCLC	1/2	Active, not recruiting
Chemotherapy drugs	NCT04194125	[^177^Lu]Lu-DOTATOC + Capecitabine and Temozolomide	25	non-resectable and/or GEP-NET G1and G2	2	Unknown
	NCT02736500	[^177^Lu]Lu-DOTATATE + Capecitabine	39	GEP-NET	1/2	Completed
	NCT05247905	[^177^Lu]Lu-DOTATOC + Capecitabine & temozolomide	31	pNET	2	Active, not recruiting
	NCT02358356	[^177^Lu]Lu-DOTATOC + Capecitabine & temozolomide	75	mNET	2	Completed
	NCT07185672	[^177^Lu]Lu-DOTATOC + Capecitabine & temozolomide	162	GEP-NET	3	Recruiting
	NCT05387603	[^177^Lu]Lu-DOTATOC + Capecitabine	300	GEP-NEN	3	Recruiting
	NCT04234568	[^177^Lu]Lu-DOTATATE + Triapine	33	NET	1	Completed
	NCT05724108	[^177^Lu]Lu-DOTATATE + Triapine	94	mNET	2	Recruiting
Epigenetic drug	NCT05178693	[^177^Lu]Lu-DOTATATE + ASTX727	27	NET	1	Recruiting
Endocrine therapeutic	NCT05884255	[^177^Lu]Lu-DOTATATE + LA TOC/High-dose LA TOC	220	GEP-NET	3	Recruiting
	NCT06855095	[^177^Lu]Lu-DOTATATE + LA SSTAs	39	NET G1 and G2	2/3	Recruiting
	NCT06663072	[^177^Lu]Lu-DOTATATE + Fulvestrant	25	pNET	1	Recruiting
External beam radiation (EBR)	NCT07150546	[^177^Lu]Lu-DOTATATE + EBR	15	Large GI NET	1	Recruiting
	NCT05109728	[^177^Lu]Lu-DOTATATE + EBR and Temozolomide	65	Recurrent Glioblastoma	1	Active, not recruiting

**Table 4:** Preclinical in vivo studies evaluating enhancement of [^177^Lu]Lu-DOTATATE therapy.

Class	Mechanism of Action	Drug	Animal Model	Results	Reference
PARP inhibitors	Inhibit DNA repair mechanisms, leading to accumulation of DNA damage	Talazoparib	AR42J xenograft mouse model	Prolonged tumor growth inhibition; improved survival	Cullinane et al., 2020 [119]
DNA-PK inhibitors	Inhibit NHEJ-mediated DSB repair	Nedisertib	H1299-SSTR2 and AR42J xenografts	Improved tumor control and prolonged survival compared with PRRT alone	Waldeck et al., 2023 [120]
		AZD7648	BON and H69 xenografts	Enhanced radiosensitivity and improved therapeutic efficacy	Reuvers et al., 2023 [121]
HSP90 inhibitors	Inhibit HSP90 chaperone function, leading to degradation of client proteins and impaired cellular stress responses	Ganetespib	GOT1 human small intestine NET xenograft model	Reduction in tumor volume	Hofving et al., 2019 [122]
		Onalespib	BON xenograft mouse model	Delayed tumor doubling time; increased SSTR2 expression	Lundsten et al., 2020. [123]
mTOR inhibitors	Inhibit PI3K/AKT/mTOR signaling, reducing proliferation and survival signaling	Everolimus (RAD001)	CA20948 rat pancreatic NET xenograft	No anti-tumor benefit; increased metastatic progression observed in non-cured animals	Pool et al., 2013 [124]
		Everolimus (RAD001)	CA20948 rat pancreatic NET xenograft	No improvement compared with PRRT alone; metastatic progression observed in animals not achieving complete remission	Bison et al., 2014 [125]
		Everolimus (RAD001)	AR42J rat pancreatic NET xenograft	No additional anti-tumor effect beyond PRRT alone; acceptable toxicity profile	Zellmer et al., 2022 [126]
Tyrosine kinase inhibitors *	Inhibit receptor tyrosine kinase signaling pathways involved in tumor growth and angiogenesis	Vandetanib (+EBR)	GOT2 human MTC xenograft	Reduction in tumor volume; prolonged time to progression	Sandblom et al., 2020 [127]
		Cabozantinib (+EBR)	GOT2 human MTC xenograft	Reduction in tumor volume; delayed tumor regrowth	Sandblom et al., 2020 [127]
Hedgehog inhibitors	Inhibit Hedgehog signaling pathway involved in tumor growth and progression	Sonidegib	GOT1 human small intestine NET xenograft	Reduction in tumor volume; prolonged time to progression; altered gene expression (e.g., *PDGFRA*)	Spetz et al., 2017 [128]
NAMPT inhibitors	Deplete intracellular NAD+, impair energy metabolism and cellular stress responses	GMX1778 (CHS 828)	GOT1 human small-intestinal NET xenograft	Enhanced anti-tumor effect of [^177^Lu]Lu-DOTATATE; prolonged progression-free survival compared with monotherapy	Elf et al., 2017 [129]
Chemotherapeutic agents	Inhibition of DNA synthesis	Gemcitabine	GOT2 medullary thyroid carcinoma xenograft	Prolonged time to progression compared with monotherapy; gemcitabine enhanced radiation response	Sandblom et al., 2019 [130]
p53 stabilizers	Stabilize p53 by inhibiting its degradation, enhancing DNA damage response and apoptosis	VIP116	Neuroblastoma xenografts	Improved tumor response and progression-free survival; increased complete remissions; no significant nephrotoxicity	Berglund et al., 2024 [131]

* Evidence derived from EBR, not [^177^Lu]Lu-DOTATATE.

**Table 5:** Results from in vitro studies on combination strategies to enhance [^177^Lu]Lu-DOTATATE therapy in NET.

Class	Mechanism of Action	Drug	Cell Type	Results	Reference
PARP inhibitors	Inhibit DNA repair mechanisms, leading to accumulation of DNA damage (DSB)	Olaparib	CA20948, U2OS	Increased uptake of [^177^Lu]Lu-DOTATATE; reduced proliferation; increased DNA damage	Nonnekens et al., 2016 [132]
		DHQ, PJ-34, ABT-888 (Veliparib)	BON, H727	Increased cytotoxicity; increased apoptosis; cell cycle arrest	Purohit et al., 2018 [133]
		Talazoparib	AR42J	Increased DNA double-strand breaks	Cullinane et al., 2020 [119]
		Olaparib, rucaparib	H69, H446	Increased DNA damage and PRRT potency	Rauch et al., 2024 [134].
DNA-PK inhibitors	Inhibit NHEJ-mediated DSB repair	Nedisertib, AZD7648	H1299-SSTR2, AR42J	Increased sensitivity to [^177^Lu]Lu-DOTATATE and reduced cell survival	Waldeck et al., 2023 [120]
		AZD7648	BON, H69, GOT1	Enhanced PRRT-induced cytotoxicity	Reuvers et al., 2023 [121]
HSP90 inhibitors	Inhibit HSP90 chaperone function, leading to degradation of client proteins and impaired cellular stress responses	Onalespib	BON, H727, H460 (monolayer and spheroids)	Synergistic effects with increased apoptosis in BON and H727 spheroids	Lundsten et al., 2019 [135]
		Onalespib	BON (ex vivo autoradiography)	No change in uptake or spatial distribution of [^177^Lu]Lu-DOTATATE	Lundsten et al., 2020 [123]
		Ganetespib	GOT1, BON-SSTR2 NET models	Enhanced radiation-induced cytotoxicity; radiosensitization mediated by proteotoxic stress rather than increased DNA damage	Engbers et al., 2025 [136]
mTOR inhibitors	Inhibit PI3K/AKT/mTOR signaling and cellular proliferation	Everolimus (RAD001)	BON	Anti-proliferative effects and growth inhibition	Zitzmann et al., 2007 [137]
Chemotherapeutic agents	Ribonucleotide reductase inhibition; S-phase accumulation	Hydroxyurea, gemcitabine, triapine	U2OS+SSTR2A, BON1	Increased SSTR2A expression, increased [^177^Lu]Lu-DOTATATE uptake, enhanced PRRT-induced cytotoxicity	Cheng et al., 2024 [138]
p53 stabilizers	Stabilize p53 by inhibiting its degradation, enhancing DNA damage response and apoptosis	VIP116	IMR-32, SK-N-AS, N2a, LU-NB-1, LU-NB-2 (monolayer and spheroids)	Synergistic effects with increased apoptosis in IMR-32 spheroids	Lundsten et al., 2021 [139]
DNA methyltransferase inhibitors (DNMTis)	Modify epigenetic regulation and gene expression, including increased SSTR expression and altered DNA repair	Decitabine + 5-FU	BON, QGP-1	Increased radiosensitivity, apoptosis, SSTR2 expression and radioligand binding	Jin et al., 2019 [140]
		5-aza2′-deoxycytidine (5-aza-dC)	SSTR2-transfected HEK293	Increased uptake and therapeutic effect of [^177^Lu]Lu-DOTATATE	Kotzerke et al., 2022 [141]
HDAC inhibitors	Modify epigenetic regulation and gene expression, including increased SSTR expression and altered DNA repair.	VPA	SSTR2-transfected HEK293	Increased uptake and therapeutic effect of [^177^Lu]Lu-DOTATATE	Kotzerke et al., 2022 [141]
BET inhibitors *	Epigenetic/transcriptional regulation	I-BET151	GOT1, P-STS	Synergistic reduction in cell viability in both siNET cell lines	Hofving et al., 2019 [122]

DHQ = 1,5-dihydroxyisoquinoline. * Evidence derived from EBR, not [^177^Lu]Lu-DOTATATE.

**Table 6:** Characteristics of tumor cell lines and experimental models used in NET-related PRRT research.

Cell Line	Origin (Species)	Tumor Type	SSTR Expression	Exocrine/Endocrine	References
AR42J	Rat	Pancreatic tumor	SSTR1,2,3,5	Exocrine	[142]
At-T20	Mouse	Pituitary tumor	SSTR2,5	Endocrine	[143]
BON/BON-1	Human	Pancreatic NET	SSTR2,3,5	Endocrine	[144]
Capan-2	Human	Pancreatic adenocarcinoma	SSTR2,5	Exocrine	[145]
CA20948	Rat	Pancreatic tumor	SSTR2	Exocrine	[146]
CM	Human	Pancreatic insulinoma	SSTR2	Endocrine	[147]
GOT1	Human	Small intestinal NET (siNET)	SSTR2	Endocrine	[148]
GOT2	Human	Medullary thyroid carcinoma (MTC)	SSTR2	Endocrine	[149,150]
H69	Human	Small cell lung cancer	SSTR1,2	-	[151]
H727	Human	Bronchopulmonary NET	Low SSTR2 expression	Endocrine	[152]
H-STS	Human	Small intestinal NET (siNET)	Low SSTR2,3 expression	Endocrine	[153]
KRJ-I	Human	Small intestinal NET (siNET)	Low SSTR2,3 expression	Endocrine	[154]
L-STS	Human	Small intestinal NET (siNET)	Low SSTR2,3 expression	Endocrine	[153]
MZ-CRC-1	Human	Medullary Thyroid Cancer	SSTR2	Endocrine	[155]
P-STS	Human	Small intestinal NET (siNET)	Low SSTR2,3 expression	Endocrine	[153]
QGP-1	Human	Pancreatic NET	Low SSTR2 expression	Endocrine	[156]
TT	Human	Medullary Thyroid Cancer	SSTR2	Endocrine	[157]
TtT-97	Mouse	Pituitary tumor	Not reported	Endocrine	[158]

**Table 7:** Overview of strategies for enhancement of [^177^Lu]Lu-DOTATATE therapy.

Strategy	Method	In Vitro	Preclinical In Vivo	Clinical
**Increasing tumor targeting and receptor availability**				
EBR-induced SSTR modulation	EBR	Increased SSTR expression and uptake in several models	Increased uptake after EBR in tumor models	NA
PRRT-induced SSTR modulation	PRRT priming/fractionation	Fractionation enhances radiobiological effects	Higher tumor uptake and absorbed dose, delayed tumor growth	Limited clinical observations (case reports); no controlled clinical studies
SSTA-mediated SSTR modulation	TOC, pasireotide (dose- and time-dependent)	Down- or upregulation depending on dose/exposure	Increased binding under certain conditions	Variable effects on tumor uptake; no consistent improvement demonstrated
Hormonal SSTR regulation	Thyroid hormone, EGF, tamoxifen	Increased SSTR expression in several cell lines	NA	NA
Epigenetic SSTR modulation	DNMTi, HDACi (e.g., 5-aza-dC, VPA)	Increased SSTR expression and uptake	Increased uptake in some models	Early phase clinical trials ongoing for SSTR upregulation strategies (no efficacy data yet)
Chemotherapy-induced SSTR modulation	Temozolomide, gemcitabine, triapine	Increased SSTR expression and uptake in some models	NA	NA
**Preventing repair of radiation-induced damage**				
PARP inhibitors	Olaparib, Talazoparib, Veliparib	Increased DNA damage and apoptosis	Enhanced tumor control and survival	Phase I clinical data demonstrate feasibility and manageable toxicity
DNA-PK inhibition	Nedisertib (peposertib), AZD7648	Increased sensitivity to PRRT through inhibition of NHEJ-mediated DNA repair	Improved tumor control and survival in NET xenograft models	Phase Ib clinical trial ongoing
**Suppressing cellular stress adaptation and survival**				
HSP90 inhibitors	Ganetespib, Onalespib	Enhanced apoptosis and radiosensitivity; radiosensitization linked to proteotoxic stress and pleiotropic pathway disruption	Reduced tumor growth and increased doubling time	No clinical studies with PRRT combinations reported
mTOR inhibition	Everolimus, temsirolimus	Reduced proliferation and tumor cell growth	Mixed results; no clear enhancement of PRRT efficacy in CA20948 or AR42J NET models	Clinical feasibility demonstrated; ongoing trials
TK inhibitors	Vandetanib, cabozantinib	NA	Improved tumor control in combination with EBR	TKIs effective as monotherapy in NETs; no clinical PRRT combination data available
Hh inhibitors	Sonidegib	NA	Reduced tumor growth, delayed regrowth, altered NF-κB and PDGF signaling	NA
NAMPT inhibitors	GMX1778	Reduced tumor cell viability through NAD+ depletion and impaired stress response capacity	Improved anti-tumor response and prolonged progression-free survival in GOT1 xenografts	NA
**Promoting tumor cell elimination**				
Immunotherapy	PD-1/PD-L1 and CTLA-4 inhibitors	Enhanced antigen presentation, immune activation, and tumor cell recognition after radiation exposure	Increased cytotoxic immune-cell recruitment and improved therapeutic efficacy when combined with [^177^Lu]Lu-DOTATATE in NET models	Early clinical studies demonstrate feasibility; multiple ongoing phase I-II trials
Chemotherapeutic agents	Gemcitabine + [^177^Lu]Lu-DOTATATE/EBR	NA	Improved tumor control and time to progression; stronger effects with EBR	No clinical studies combining with PRRT; gemcitabine used clinically in NETs
	Capecitabine, 5-FU, temozolomide, gemcitabine, triapine	Increased SSTR expression and radioligand uptake; radiosensitization through DNA synthesis inhibition, S-phase accumulation, and impaired DNA repair	Improved tumor control and prolonged time to progression in selected models	Most clinically mature combination strategy; multiple positive studies and ongoing randomized trials, although not all studies have demonstrated superiority over PRRT alone
p53 stabilization	VIP116	Stabilization of p53; enhanced cell-cycle regulation and apoptosis	Improved PRRT response in spheroids and in vivo neuroblastoma models	NA
Epigenetic modifiers	DNMTi, HDACi, BETi	Increased apoptosis and radiosensitivity	NA	NA

NA = Not available, TK = tyrosine kinase, Hh = Hedgehog, NAMPT = Nicotinamide phosphoribosyltransferase.

## Data Availability

No new data were created or analyzed in this study. Data sharing is not applicable to this article.
